# Exploration of the sputum methylome and omics deconvolution by quadratic programming in molecular profiling of asthma and COPD: the road to sputum omics 2.0

**DOI:** 10.1186/s12931-020-01544-4

**Published:** 2020-10-19

**Authors:** Espen E. Groth, Melanie Weber, Thomas Bahmer, Frauke Pedersen, Anne Kirsten, Daniela Börnigen, Klaus F. Rabe, Henrik Watz, Ole Ammerpohl, Torsten Goldmann

**Affiliations:** 1 LungenClinic Grosshansdorf, Großhansdorf, Germany; 2 Airway Research Center North (ARCN), Member of the German Center for Lung Research (DZL), Großhansdorf, Germany; 3 Department of Internal Medicine I, Pneumology, University Hospital Schleswig-Holstein, Campus Kiel, Kiel, Germany; 4 Department of Oncology, Hematology and BMT with Section Pneumology, University Medical Center Hamburg-Eppendorf, Hamburg, Germany; 5 Program in Applied and Computational Mathematics, Princeton University, Princeton, NJ USA; 6 Pulmonary Research Institute at LungenClinic Grosshansdorf, Großhansdorf, Germany; 7 Bioinformatics Core Unit, University Medical Center Hamburg-Eppendorf, Hamburg, Germany; 8 Institute of Human Genetics, University Medical Center Ulm, Ulm, Germany; 9 Research Center Borstel, Pathology, Borstel, Germany

**Keywords:** Sputum, Omics, Transcriptome, Methylome, Deconvolution, RNA, Degradation, Biobanking, Asthma, COPD

## Abstract

**Background:**

To date, most studies involving high-throughput analyses of sputum in asthma and COPD have focused on identifying transcriptomic signatures of disease. No whole-genome methylation analysis of sputum cells has been performed yet. In this context, the highly variable cellular composition of sputum has potential to confound the molecular analyses.

**Methods:**

Whole-genome transcription (Agilent Human 4 × 44 k array) and methylation (Illumina 450 k BeadChip) analyses were performed on sputum samples of 9 asthmatics, 10 healthy and 10 COPD subjects. RNA integrity was checked by capillary electrophoresis and used to correct in silico for bias conferred by RNA degradation during biobank sample storage. Estimates of cell type-specific molecular profiles were derived via regression by quadratic programming based on sputum differential cell counts. All analyses were conducted using the open-source R/Bioconductor software framework.

**Results:**

A linear regression step was found to perform well in removing RNA degradation-related bias among the main principal components of the gene expression data, increasing the number of genes detectable as differentially expressed in asthma and COPD sputa (compared to controls). We observed a strong influence of the cellular composition on the results of mixed-cell sputum analyses. Exemplarily, upregulated genes derived from mixed-cell data in asthma were dominated by genes predominantly expressed in eosinophils after deconvolution. The deconvolution, however, allowed to perform differential expression and methylation analyses on the level of individual cell types and, though we only analyzed a limited number of biological replicates, was found to provide good estimates compared to previously published data about gene expression in lung eosinophils in asthma. Analysis of the sputum methylome indicated presence of differential methylation in genomic regions of interest, e.g. mapping to a number of human leukocyte antigen (HLA) genes related to both major histocompatibility complex (MHC) class I and II molecules in asthma and COPD macrophages. Furthermore, we found the SMAD3 (SMAD family member 3) gene, among others, to lie within differentially methylated regions which has been previously reported in the context of asthma.

**Conclusions:**

In this methodology-oriented study, we show that methylation profiling can be easily integrated into sputum analysis workflows and exhibits a strong potential to contribute to the profiling and understanding of pulmonary inflammation. Wherever RNA degradation is of concern, in silico correction can be effective in improving both sensitivity and specificity of downstream analyses. We suggest that deconvolution methods should be integrated in sputum omics analysis workflows whenever possible in order to facilitate the unbiased discovery and interpretation of molecular patterns of inflammation.

## Background

Respiratory research has greatly benefited from the application of molecular high-throughput (“omics”) technologies [1, 2]. Significant contributions could be made to the understanding of chronic-inflammatory respiratory disease, ranging from phenotyping and classifying disease to modeling therapy responses [3–7]. Amongst other materials, induced sputum has proven to be very valuable for the molecular profiling of both bronchial asthma and chronic obstructive pulmonary disease (COPD) [3, 6, 8–11]. With growing availability of computational infrastructure and analysis platforms, multi-omics approaches gained attractivity and have been applied successfully [2, 12–14]. In this context, epigenetic analyses, such as DNA methylation profiling, have contributed to the molecular characterization of inflammation in asthma and COPD [15–21]. So far, however, methylation analyses of sputum samples have been limited to subsets of loci, e.g. by the means of methylation-sensitive polymerase chain reaction (PCR), in cancer research [22–24]. The use of whole-genome methylation analyses of sputum for the molecular profiling of asthma or COPD has not been evaluated yet.

Sputum samples contain a mixture of immune cells (mainly alveolar macrophages, neutrophils, eosinophils and lymphocytes), but also contaminating cells (such as ciliated epithelium from the airways and squamous epithelium from the pharyngeal and oral region). The relative abundancy of cell types varies substantially and can be used to distinguish disease subgroups such as “T2 high” from “T2 low” types in asthma by eosinophil counts [25, 26]. This variability, in turn, has potential to confound whole-sputum omics analyses [27]. Previously, methods such as fluorescence activated cell sorting (FACS) [28], gradient centrifugation [29, 30] or selection by cellular adherence [31] have been used to purify certain cell types from blood and bronchoalveolar lavage (BAL) samples in asthma and COPD. Due to the corresponding procedural and financial effort, however, the implementation of such methods becomes complicated in large-scale settings. Apart from physical cell separation, attempts to correct for cellular composition in silico have been made in omics analyses of BAL [32] and blood samples [33]. Recently, a reference-based transcriptomic method thought to be less sensitive to sputum composition bias has been suggested for use in asthma research [10].

However, the aforementioned approaches do not allow to infer cell-type specific molecular profiles from mixed-cell data and, so far, the high-throughput molecular analysis of mixed-cell sputum samples has generally been limited to be used as a molecular “fingerprint” to describe inflammatory processes.

Over the last years, tailored in silico methods, so-called deconvolution algorithms, have been designed to solve the problem of inferring cell type-specific omics profiles from mixed-cell data [34–41]. These methods have been primarily developed and evaluated on blood, brain and cancer data sets but exhibit a strong potential to be of avail for omics analyses of sputum and other respiratory mixed-cell materials.

In this exploratory and methodology-oriented study, we examine the applicability of sputum whole-genome methylation analysis in molecular profiling of asthma and COPD. Furthermore, we provide insight into how in silico RNA quality correction can benefit the transcriptome analysis of sputum samples from long-term storage and, for the first time, apply a deconvolution method based on linear regression by quadratic programming to infer cell type-specific omics profiles from mixed-cell sputum data.

## Materials and methods

### Sputum samples

Sputum samples were obtained from biomaterial depositories of the German prospective cohort studies ALLIANCE [42] (asthma and controls) and COSYCONET [43] (COPD) at the LungenClinic Grosshansdorf, Germany. To evaluate the applicability of methylation profiling to sputum samples from (long-term) biobank storage, we focused on samples collected during early recruiting periods (11/2013–05/2015).

Asthma samples were selected from 9 subjects representing a phenotype with eosinophilic (type 2) inflammation and overall good disease control at the time of sample collection based on sputum and blood eosinophil count (≥ 2% eosinophils in sputum differential cell count and/or ≥ 400 eosinophils/µL in blood differential) as well as total asthma control test (ACT) score (≥ 20 points). In addition, subjects had to be non-smokers (smoking cessation > 12 months before sample collection) with a neglectable smoking history (2/9 subjects, maximum of 5 packyears). No subject was treated with biologics, antihistamines or oral corticoids at the time of sputum collection. Out of the 9 subjects, 7 had a proven allergy (pollinosis, food allergy and/or atopic dermatitis). A total of 5 subjects presented with severe asthma (defined as requiring high doses of inhaled corticosteroids with > 500 µg fluticasone equivalent per day) and 4 presented with a mild-to-moderate type (≤ 500 µg per day).

A total of 10 COPD samples was selected from subjects that had not experienced any moderate or severe exacerbation (defined as requiring use of oral corticoids or inpatient hospital treatment) for ≥ 12 months as well as had successfully accomplished smoking cessation for ≥ 12 months. Of the 10 selected subjects, 7 had moderate (GOLD 2) and 3 had severe COPD (GOLD 3).

Healthy controls (10 samples) were defined as subjects without any history of pulmonary or systemic-inflammatory disease, allergies or respiratory tract infection within the last 12 months. None of the selected control subjects had any smoking history. Descriptive statistics can be found in Table 1.

**Table 1:** Descriptive statistics of study subjects

	Age (years)	Gender (male/female)	Smoking history (PY)	Daily ICS (µg FE)
	Mean ± SD (min/max)		Mean ± SD (min/max)	Mean ± SD (min/max)
Asthma n = 9	59 ± 14 (35/76)	6/3	1 ± 2 (0/5)	511 ± 388 (0/1000)
COPD n = 10	68 ± 10 (44/77)	9/1	38 ± 21 (15/80)	235 ± 206 (0/500)
Controls n = 10	44 ± 20 (19/76)	7/3	0	0

*PY* pack years, *ICS* inhaled corticosteroid, *FE* fluticasone equivalent

**Fig. 1:**
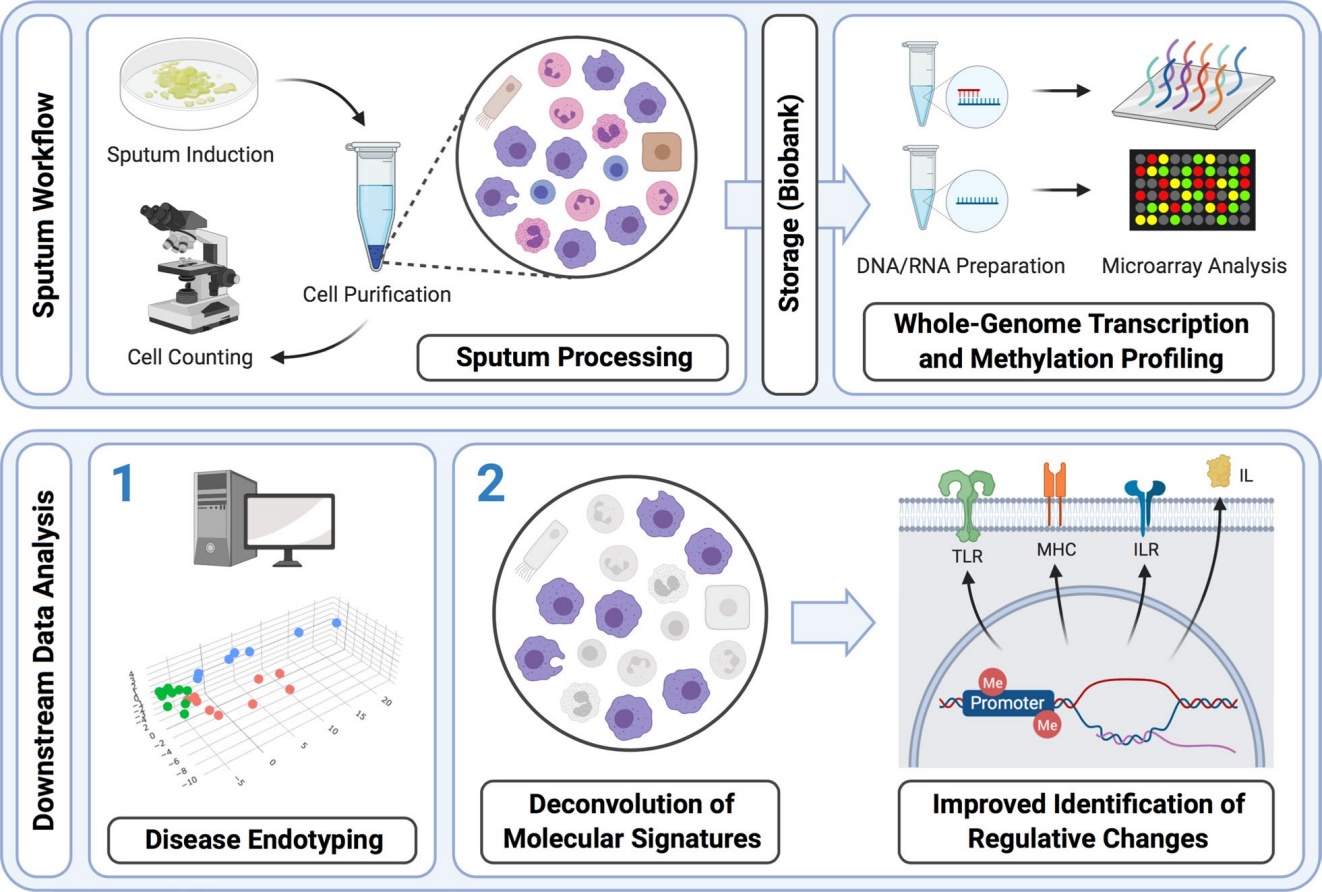
Graphical abstract. Induced sputum, containing a variety of inflammatory cells, exhibits potential to directly reflect inflammatory processes in the lower airways. Progress in understanding the underlying mechanisms has been made by supplying sputum samples to high-throughput molecular analyses, primarily transcriptomics. To date, these have provided valuable insights to disease mechanisms and have led to differentiation of molecular endotypes (1) that are associated with distinct clinical presentations. However, most high-throughput analyses of sputum samples are prone to substantial bias by variation in cellular composition. Here, we introduce an unbiased deconvolution approach to sputum omics analysis in order to improve the identification of molecular patterns and dysregulation (2). Furthermore, were provide an example that sputum analysis can be extended by whole-genome methylation profiling to broaden the view on molecular mechanisms of pulmonary inflammation. Created with BioRender.com

For a graphical representation of the overall workflow, see Fig. 1.

Details about sputum induction and processing are provided in Additional file 1. Differential sputum cell counts (alveolar macrophages, neutrophils, eosinophils, lymphocytes, monocytes, ciliated epithelium and squamous cells) were performed on Diff-Quick-stained slides by two independent evaluators, each of whom evaluated a total of 400 cells per slide.

Samples were either stored in RLT Plus extraction buffer (proprietary buffer by Qiagen, Hilden, Germany) at − 80 °C [9], or preserved via the HOPE technique (Hepes-glutamic acid buffer-mediated organic solvent protection effect) by incubation with HOPE medium (DCS innovative diagnostic systems, Hamburg, Germany), followed by embedding in low-melting paraffin and subsequent storage at 4 °C [44]. The HOPE technique is a preservation technique originally developed for tissue samples in pathology diagnostics and research to allow for a variety of processing and analysis protocols without the constraints imposed by conventional formalin fixation [45]. Previous studies have demonstrated that HOPE-preserved material can successfully be processed to retrieve nucleic acids suitable for omics analysis [45, 46] and that the technique can be transferred to sputum samples [44] as well as bronchoalveolar lavage fluid [47].

### Extraction of nucleic acids

From HOPE-preserved, paraffin embedded samples, sputum cells were extracted by cutting slices on a microtome (using alcohol- and heat-sterilized, RNase-free blades) which were deparaffinized subsequently by incubation with xylene (2 × 10 min) and ethanol (2 × 10 min), followed by a drying step using a vacuum centrifuge before addition of RLT Plus lysis buffer [44, 47]. Sputum samples stored in RLT buffer were thawed on ice. DNA and RNA were simultaneously extracted using the AllPrep Micro Kit (Qiagen) following the manufacturer’s instructions. Total DNA and RNA yield were measured on a NanoDrop spectrophotometer (Thermo Fisher Scientific, Waltham, MA, USA). RNA integrity was determined on a BioAnalyzer system (Agilent Technologies, Waldbronn, Germany). For optimal electrophoresis resolution, the RNA 6000 Pico Kit (Agilent) was used after adjusting aliquots of RNA extracts to the maximum input RNA concentration. Samples with RIN < 3 (RNA Integrity Number) were excluded from further processing for microarray analysis which applied to a total of 4 HOPE-preserved samples, limiting the total number of successfully hybridized expression arrays to 25 out of 29 (9 asthma, 7 COPD, 9 controls).

### Transcription microarray analysis

Extracted total RNA was processed with Agilent’s Low Input Quick Amp Labeling Kit. Labeled complementary RNA (cRNA) was purified using the RNeasy Mini Kit (Qiagen) and 1650 ng of labeled cRNA per sample was hybridized to Agilent Human GE 4 × 44 K v2 arrays. All steps were performed according to the manufacturers’ standard instructions. Hybridized arrays were scanned with an Agilent SureScan microarray scanner (5 µm resolution, default settings) and scan images were analyzed with Agilent’s Feature Extraction Software (version 11.5.1, default parameters, protocol GE1_1105_Oct12). All hybridized arrays passed the manufacturer’s standard quality controls.

### Methylation microarray analysis

Genomic DNA was bisulfite converted utilizing the EZ DNA Methylation kit (ZymoResearch, Irvine, CA, USA) following the manufacturer’s instructions. Converted DNA was further processed and hybridized to Infinium HumanMethylation 450 k BeadChips (Illumina Inc., San Diego, CA, USA) following the standard Illumina workflow. Hybridized chips were scanned with an Illumina iScan system on default settings. All chips passed the manufacturer’s standard quality controls as well as further quality controls applied within the downstream in silico analysis. Due to a technical error, three samples were lost during processing, limiting the total number of samples from which methylation data was available to 26 (9 asthma, 10 COPD, 7 controls).

### Data analysis

Downstream data analysis was entirely performed using the open-source R/Bioconductor software framework [48] (https://www.r-project.org, https://www.bioconductor.org). Supplementary methodological information as well as a comprehensive list of utilized software packages is provided in Additional file 1: Table S3). All annotation data used throughout this study was entirely based on the human genome version hg19 in accordance with the utilized array platforms. Transcriptome and methylome data have been made publicly available via NCBI’s Gene Expression Omnibus [49] (see “Availability of data and materials”).

Methylation array data was imported, annotated and processed by stratified quantile normalization utilizing the *minfi* package [50]. Individual CpG loci were filtered before further analysis by detection p values (threshold p = 0.01), mapping to sex chromosomes (X and Y chromosomes were excluded), affection by single nucleotide polymorphisms (SNPs) as well as potential for cross hybridization based on data published by Chen et al. [51]. Hereafter, a total of 429,236 CpGs (of initial 485,512) was further analyzed.

For gene expression data, median foreground signals were background corrected by subtracting the mean background signals (“minimum” method) via the *limma* package [52]. Quantile normalization was applied and control probes were filtered out. We used flagging information generated by the Feature Extraction Software (Agilent) to further exclude probes that were classified as non-uniform, saturated or feature population outlier in any of the arrays. Furthermore, at least 50% of features per array probe in any of the sample groups had to be classified as being found as well as being positive and significant to be kept in the data set. Replicate probes were averaged and further analysis steps were carried out on probe level (27,380 array probes of 44,495 initial feature reads retained).

### Differential expression/methylation analysis

Differentially expressed genes (DEGs) as well as differentially methylated CpGs (differentially methylated positions, DMPs) were determined via *limma* comparing disease entities to healthy controls (asthma vs. controls and COPD vs. controls).

For DEGs, statistics were calculated on log_2_-transformed expression values with Benjamini–Hochberg (BH)-adjusted p value < 0.05 and absolute log_2_-fold change (log_2_FC) ≥ 1.5 as statistical significance cutoffs. To remove redundancy from the data set and to simplify the biological interpretation of results, DEGs were filtered for well-annotated transcripts (based on available ENSEMBL and RefSeq annotation) and the most significantly differentially expressed transcript per gene was reported.

DMPs were determined on the beta value scale and considered statistically significant at a BH-adjusted p value < 0.05 and delta beta ≥ 0.1.

### Deconvolution of cell type-specific expression and methylation

Generally speaking, the deconvolution we applied is based on the idea that estimates for cell type-specific expression/methylation can be derived by finding (optimizing) estimates that, given the relative cell counts for each sample, best match the observed (measured) mixed-cell expression/methylation. This poses a classical regression problem which gets complicated by the circumstance that both expression and methylation have biological limits (e.g., expression cannot be negative) that must not be violated by the mathematical optimization process in order to get biologically possible and meaningful results. In technical detail, we performed regression-based deconvolution (by quadratic programming) using the differential sputum cell counts as predictor variables and the measured mixed-cell omics profiles (expression/methylation) as response variables in the underlying linear models. To allow for linear combinability of the input data, expression values had to be analyzed on the linear (instead of log_2_-transformed) and methylation values on the beta value scale for the purpose of deconvolution. The general performance of a multiple linear regression approach was evaluated by fitting models with built-in functions of R (*stats* R core package). The estimation was carried out by quadratic programming (QP), allowing us to specify biological constraints under which the regression parameters were estimated. This approach had previously been successfully applied to methylation and gene expression data [36, 38]. A detailed mathematical description is provided in Additional file 1. In short, we utilized the *quadprog* R package (https://cran.r-project.org/web/packages/quadprog/index.html) which implements the dual method of Goldfarb and Idnani to solve quadratic programs [53]. Estimation was performed for each sample group separately, methylation estimates were constrained to the interval between 0 and 1, expression estimates to the dynamic range of the array. We estimated the standard errors of the estimates following a standard approach in regression analysis as previously applied [38]. Comparisons of methylation and expression estimates across disease groups was followingly carried out with a Welch modified two-sample (unequal variance) t-test. Taking into account that, due to the distribution of the analyzed methylation and expression values, one of the core assumptions of parametric testing (normality) was likely violated, we applied more stringent p cutoffs to assign statistical significance: DMPs were considered statistically significant at a BH-adjusted p < 0.001 and delta beta ≥ 0.1. For DEGs, the BH-adjusted p cutoff was set to 0.005 at a log_2_FC ≥ 1.5.

### Identification of DMRs

Differentially methylated regions (DMRs) were identified via *DMRcate* [54]. For the mixed-cell methylation data, the overall false discovery rate (FDR) was set to 0.05. For the analysis of the deconvolved estimates of cell type-specific methylation, the overall FDR was set to 0.001 (as for the identification of individual DMPs). DMRs had to contain at least one CpG with delta beta > 0.1 to be considered for further analysis. Mapping of DMRs to genomic regions of interest was performed to promoter (defined as up to 1500 base pairs upstream of the transcription start site) and gene body regions with a minimum required overlap of 200 base pairs.

### GO and KEGG enrichment

Analyses for enrichment in Gene Ontology (GO) terms [55] and Kyoto Encyclopedia of Genes and Genomes (KEGG) pathways [56] were carried out via the *clusterProfiler* package [57]. Hypergeometric overrepresentation tests (cutoff p < 0.1, cutoff q < 0.2) were performed with custom backgrounds based on the array designs after probe filtering.

## Results

### Sample and data evaluation

**Fig. 2:**
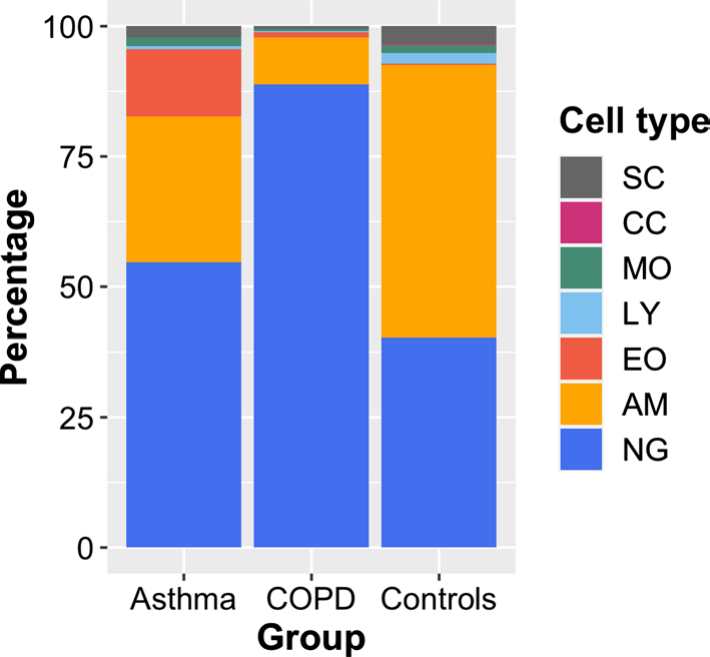
Mean cellular composition of sputum samples. *AM* alveolar macrophages, *NG* neutrophil granulocytes, *EO* eosinophils, *LY* lymphocytes, *MO* monocytes, *CC* ciliated cells (respiratory epithelium), *SC* squamous cells

Asthma samples were largely composed of eosinophils, alveolar macrophages and neutrophils. Whilst eosinophils were nearly absent in COPD and control samples, COPD sputum was greatly composed of neutrophils and that of healthy controls mainly of alveolar macrophages (see Table 2 and Fig. 2). Apart from the differences observed across conditions, substantial interindividual variation could be observed within the respective groups (for further information see Additional file 1: Tables S1, S2 and Figure S1.

**Table 2:** Differential cell count of sputum samples

	AM	NG	EO	LY	MO	CC	SC
Asthma n = 9	27.9 ± 21.9 (6.3/60.4)	54.7 ± 24.4 (14.1/84.8)	12.9 ± 24.5 (1.5/77.0)	0.7 ± 0.5 (0.1/1.6)	0.1 ± 0.1 (0.0/0.3)	1.6 ± 1.0 (0.5/3.3)	2.1 ± 3.4 (0.3/10.8)
COPD n = 10	9.0 ± 5.9 (1.1/21.1)	88.9 ± 6.6 (76.6/98.1)	1.0 ± 1.2 (0.0/4.0)	0.2 ± 0.3 (0.0/0.8)	0.0	0.4 ± 0.4 (0.0/1.4)	0.6 ± 0.5 (0.0/1.8)
Controls n = 10	52.3 ± 24.9 (16.3/81.3)	40.3 ± 25.1 (6.5/76.1)	0.2 ± 0.4 (0.0/1.1)	2.0 ± 2.1 (0.0/7.6)	0.2 ± 0.3 (0.0/0.9)	1.6 ± 0.8 (0.4/2.6)	3.4 ± 3.3 (0.4/10.4)

Cell proportions are reported as mean percentage ± SD (min/max)

*AM* alveolar macrophages, *NG* neutrophil granulocytes, *EO* eosinophils, *LY* lymphocytes. *MO* monocytes, *CC* ciliated cells (respiratory epithelium), *SC* squamous cells

Those samples that had been specifically preserved for nucleic acid preparation by storage in RLT extraction buffer were found to provide RNA of overall good quality (RIN ranging from 7.6 to 9.1, see Table 3). Concomitantly, HOPE-preserved samples were subject to a higher amount of RNA degradation during biobank storage (maximum RIN 5.1).

**Table 3:** RNA integrity of sputum samples supplied to gene expression analysis

	RIN	Preservation
	Mean ± SD (min/max)	(RLT/HOPE)
Asthma n = 9	6.4 ± 2.5 (3.2/8.7)	5/4
COPD n = 7	5.6 ± 1.7 (4.2/8.5)	2/7
Controls n = 9	7.4 ± 2.1 (4.2/9.1)	6/3
RLT n = 13	8.6 ± 0.4 (7.6/9.1)	13/0
HOPE n = 12	4.3 ± 0.6 (3.2/5.1)	0/12

*RIN* RNA integrity number, *RLT* preservation by storage in RLT buffer, *HOPE* preservation via HOPE-fixation technique

**Fig. 3:**
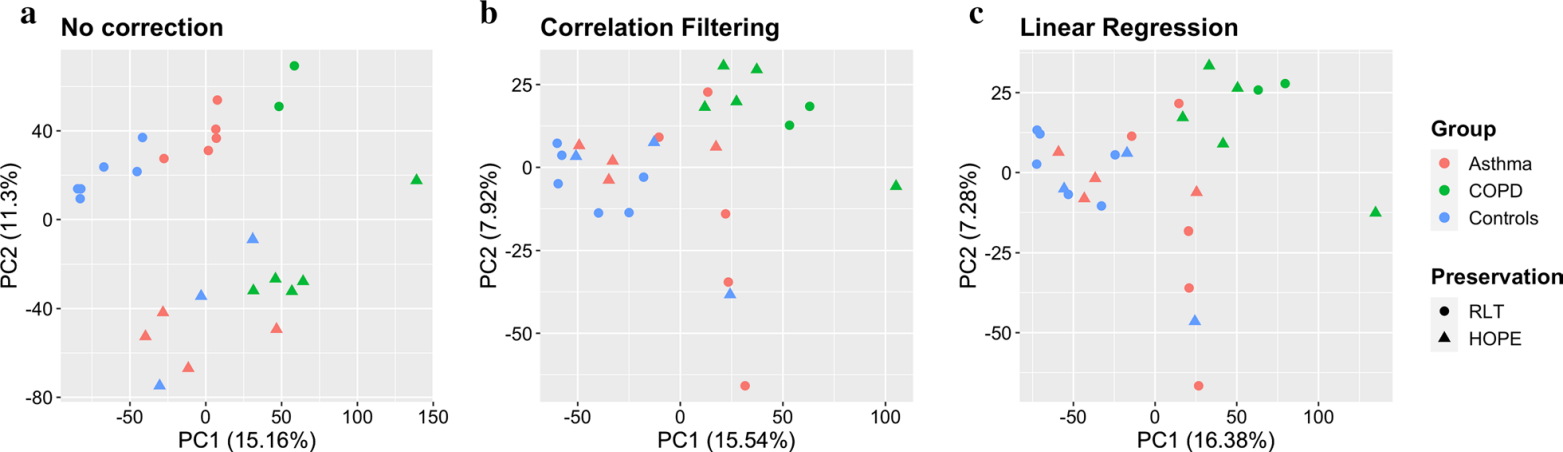
Principal component analysis of the gene expression data. Before correction for RNA degradation (**a**), after correlation filtering (**b**) and after correction by linear regression (**c**)

The amount of RNA degradation could be shown to have a substantial effect on the overall variation in the expression data set (see principal component analysis in Fig. 3a). Gene expression values were both positively and negatively correlated with RNA integrity which follows from the rank-based process of quantile normalization applied to the data (see Additional file 1: Figure S2). Excluding array probes from further analysis by the extent of correlation (correlation filtering—reducing the data set to 15,550 transcripts out of 27,380; further details are provided in Additional file 1) efficiently removed major degradation effects (Fig. 3b) but was observed to be biased towards medium-to-highly expressed transcripts (Additional file 1: Figures S4 and S5). This approach was outperformed by a correction via a linear model (for details see Additional file 1) which ensured removal of degradation bias (Fig. 3c) without reducing the number of transcripts eligible for analysis (see also Additional file 1: Figure S6).

**Fig. 4:**
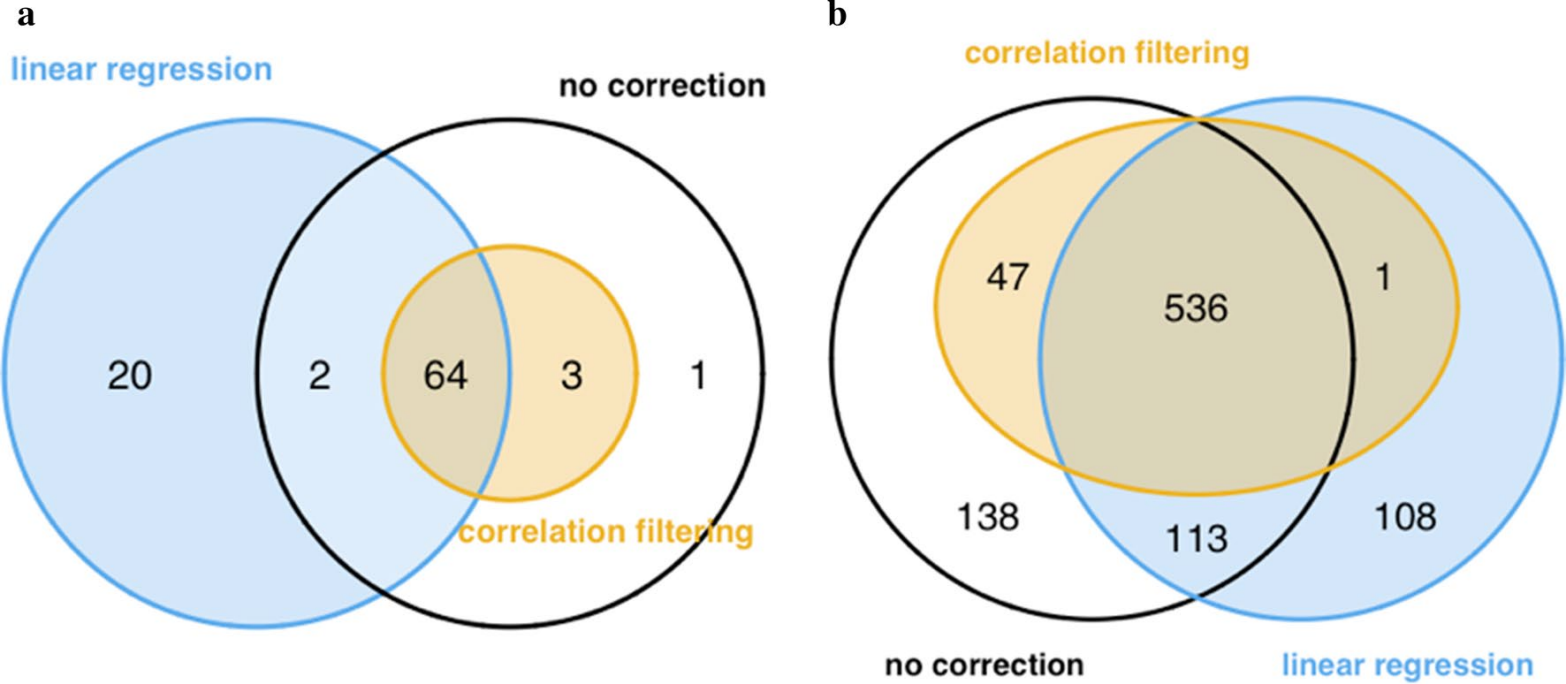
Venn diagram visualizations of differentially expressed genes (DEGs). Asthma vs. controls (**a**) and COPD vs. controls (**b**). Analyses were performed on the uncorrected, mixed-cell transcriptome dataset (white/black circle), after correction for RNA degradation by correlation filtering (yellow) and after correction by linear regression (blue)

In case RNA degradation is equally distributed among compared sample groups, it can be assumed to primarily affect analysis sensitivity (more false negatives) whilst not necessarily leading to a higher proportion of false positives. Should the extent of degradation be distributed unequally among samples (the COPD samples exhibited a higher extent of degradation than the asthma or control samples), however, RNA degradation-biased expression data can in fact be expected to lead to falsely identified DEGs (false positives). Concordantly, only a minor number of DEGs in asthma identified in the uncorrected data was discarded by correlation filtering whilst RNA integrity correction by linear regression led to identification of additional DEGs (Fig. 4a). In COPD, reduction of the data set by correlation filtering primarily resulted in a concomitant reduction of the number of DEGs, whereas the linear model performed better in discarding and identifying new DEGs (Fig. 4b). Accordingly, all further analyses were performed on expression data corrected for RNA degradation by a linear model.

**Fig. 5:**
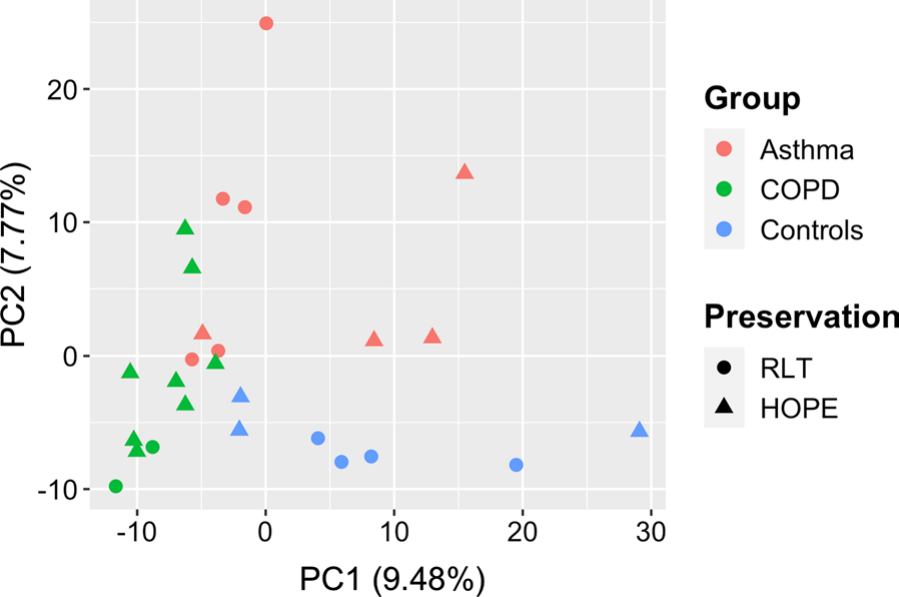
Principal component analysis of the whole-genome methylation data

The methylation data, in contrast, exhibited no major influence by the respective sample preservation method (Fig. 5). Whereas asthma and control samples did not form separate clusters among the major principal components in an unsupervised analysis of the expression data (Fig. 4c), the methylation data interestingly allowed for a better separation of control and asthma samples (Fig. 5).

### Deconvolution of cell type-specific gene expression and methylation

Since monocytes were only present in an overall very low quantity (and were not determinant in the COPD samples, see Table 2 and Additional file 1: Table S2), they were excluded from the deconvolution model ab initio. For both the expression and methylation data, estimates for macrophages, neutrophils and eosinophils (the latter in the asthma samples only) were found to be the most reliable, as inferred from the distribution of the p values associated with the respective fits of linear models (see Additional file 1: Figure S8). Consistently, the initial expression and beta value distributions were best retained in estimates for these cell types (see Additional file 1: Figures S6, S7, S9 and S10). This essentially follows from the mathematical nature of a linear model—estimation performs best for cell types that are prevalent whilst exhibiting variance across samples within a group. Strictly speaking, the estimation of macrophage profiles did not perform as well in the COPD samples as in asthma or controls. However, as the p value and expression/methylation value distributions still suggested a better performance than for the remaining cell types that could not be reliably estimated whatsoever, we decided to keep alveolar macrophages as predictor in the model fitted on the COPD data. We subsequently summarized the quantities of all other cell types and kept their sums as weighed intercepts in the models, thereby increasing the degrees of freedom of our analysis (see Additional file 1: Figures S11 to S13 and Tables S4 to S7).

Differential expression analysis of the cell type-specific estimates after deconvolution resulted in DEGs that only shared a minor proportion with those determined by analysis of the mixed-cell whole-sputum data (see Additional file 1: Figure S14). Similar observations could be made for DMPs.

DEGs identified as being upregulated in asthma in the mixed-cell analysis only (and discarded after deconvolution) clearly showed a pattern of estimated predominant expression in eosinophils (and partly neutrophils) whilst being lowly expressed in macrophages. In the background of higher sputum eosinophil counts in asthma this exemplifies how mixed-cell sputum analyses can be biased by disease-specific variation of cellular composition. For downregulated DEGs, the picture was the opposite around (see Additional file 1: Figure S15). In COPD, upregulated DEGs were estimated to be highly expressed in neutrophils and showed lower expression in macrophages in both the COPD and control samples. Though the overall expression in neutrophils seemed to be actually higher in COPD than in controls, the higher proportion of neutrophils in the COPD sputa is likely to still have had a major skewing influence. Downregulated DEGs showed a clear trend towards high estimated expression in macrophages. Similar patterns were observed for DMPs derived from the mixed-cell methylation analysis (see Additional file 1: Figure S16).

### Differential expression analysis

A comprehensive compilation of results is provided as Additional files 2, 3, 4, 5, 6, 7, 8 and 9. In total, 86 genes were found to be differentially expressed in the asthma samples via the mixed-cell analysis (84 upregulated and 2 downregulated). After deconvolution by quadratic programming, 155 DEGs were identified for alveolar macrophages (13 up, 142 down) and 552 DEGs for neutrophils (145 up, 407 down).

DEGs identified by mixed-cell analysis were enriched in Gene Ontology (GO) terms highly related to immune response and regulation (see Additional file 5), e.g. by including CXCR1 and CXCR2 (chemokine CXC-motif receptors 1 and 2) as well as IL5RA (interleukin 5 receptor alpha). After deconvolution, DEGs in macrophages continued to be highly related to immune regulation but presented a greatly different picture of involved genes (such as TLR6, Toll-like receptor 6, and CD8A, cluster of differentiation 8a) and processes. In neutrophils, though immune-related genes could be identified, such as IL4R (interleukin 4 receptor) and CXCL2 (chemokine CXC-motif ligand 2), they did not significantly enrich in GO terms at the specified significance cutoffs. Results of KEGG pathway enrichment were related to immune regulation in the mixed-cell results and in macrophages (Additional file 6). As with GO enrichment, no KEGG pathways were significantly enriched in the neutrophil gene set.

The analysis of COPD samples resulted in 758 DEGs (612 up, 146 down) in the mixed-cell data and in 39 (10 up, 29 down) and 2161 (168 up, 1993 down) DEGs in macrophages and neutrophils after deconvolution, respectively. Whereas enriched GO terms were highly related to inflammatory processes (foremost neutrophil immunity) before deconvolution (with e.g. IL6R, interleukin-6 receptor, amongst them), no enriched GO terms could be identified in macrophages after deconvolution. CXCL9, chemokine (CXC motif) ligand 9, however, was found to be among the DEGs along with MMP13 (matrix metallopeptidase 13). GO enrichment in the neutrophils’ DEGs resulted in a predominant picture of metabolic processes and regulation. Whilst enriched KEGG pathways were immunity-related in the mixed-cell analysis (e.g. containing the tumor necrosis factor, TNF signaling pathway), enrichment in macrophages, similar to the GO analysis, did not produce statistically significant results. Pathway terms significantly enriched in neutrophils were again related to metabolism, including the peroxisome and lysosome.

### Differential methylation analysis

Genes that could be associated with differentially methylated regions in asthma (mixed-cell analysis) included a small quantity of immunity-related members such as IL27RA (interleukin 27 receptor alpha), IL20 (interleukin 20) and TNF but were overall dominated by small nucleolar RNA (snoRNA) as well as small Cajal body RNA (scaRNA) genes and thereby enriched in the GO term “Cajal body” (Additional file 7). After deconvolution, DMRs found in macrophages were still largely associated with small nucleolar RNAs, but also IL23A (interleukin 23 alpha), and CCL24 (chemokine C–C motif ligand 24, previously known as eotaxin-2). GO enrichment resulted in terms largely related to regulation of development and differentiation as well as cellular interaction by adhesion. In neutrophils, amongst a number of snoRNA genes, IL5RA (interleukin receptor 5 alpha) was found to be DMR-associated. Here, GO enrichment again resulted in terms primarily associated with developmental regulation and cell adhesion. Enriched KEGG pathways could be strongly related to inflammatory processes and regulation in the mixed-cell analysis (with TNF and the HLA, human leukocyte antigen, loci HLA-DRA and HLA-DOB largely contributing to this finding), including the KEGG pathway “Asthma” (Additional file 8). After deconvolution, KEGG enrichment was limited to “Antigen processing and presentation” in macrophages but with a higher number of gene hits, comprising genes associated with both MHC (major histocompatibility complex) class I (HLA-E, HLA-F) and class II (HLA-DMA, HLA-DMB, HLA-DOA, HLA-DPA1, HLA-DPB1) as well as heat shock protein genes, amongst others. In neutrophils, no KEGG pathways were significantly enriched.

In COPD, DMR-associated genes were related to immunity by being involved in neutrophil activation as well as antigen presentation. After deconvolution, enriched GO terms mostly related to developmental regulation in both macrophages and neutrophils. However, in macrophages, the immunity-related genes IL1RN (interleukin 1 receptor antagonist) and IL20RA (interleukin receptor 20 alpha) were found to be DMR-associated. Enriched KEGG pathways were related to inflammation and immunological regulation in the mixed-cell analysis and continued to be in macrophages after deconvolution. Again, HLA loci associated both with MHC class I and II (HLA-B, HLA-E, HLA-F as well as HLA-DMA, HLA-DMB, HLA-DQA2, HLA-DRA) contributed to this finding. No pathways were significantly enriched in COPD neutrophils (see also Additional file 1: Figure S17).

### Integrative analysis

The performed deconvolution limits the applicability of some approaches for the integrative analysis of methylation and gene expression after deconvolution. E.g., a conventional correlation analysis of promoter-CpG beta values with gene expression values is not applicable to the estimates of cell-specific expression/methylation and their respective variances derived from the deconvolution. As a straightforward workaround, we decided to find overlaps between DMR-associated genes and DEGs: in the mixed-cell analysis, 1 gene was found to be both differentially expressed and methylated in asthma (see Additional file 1: Figure S18). After deconvolution, the respective quantities of DMR-associated DEGs were 23 for macrophages and 15 for neutrophils. In COPD, 74 DMRs mapped to DEGs that were identified without deconvolution. In the deconvolved data, 3 DEGs were differentially methylated in macrophages and 39 in neutrophils (see Additional file 9 for more details).

### Data comparison

We did not encounter publicly available data sets that we could subject to the deconvolution approach described here for a validation of our results, largely because detailed information about the cellular composition of samples was not reported (further details are provided in Additional file 1).

Instead, we compared our data to a transcriptome study by Esnault et al. [29] who defined a core gene set predominantly expressed by lung eosinophils in asthma by expression profiling of BAL and sputum in the context of allergen challenges that was subsequently validated in purified lung eosinophils. In good agreement, this gene set was estimated to be predominantly expressed in eosinophils in our deconvolved data (see Additional file 1: Figure S19 and accompanying information). In accordance, we found the eosinophil marker genes RNASE2 (ribonuclease A family member 2), RNASE3, SIGLEC8 (sialic acid binding Ig-like lectin 8) and IL5RA [29] as well as PRSS33 (serine protease 33) [58] being exclusively expressed in eosinophils in the deconvolved asthma data (see Additional file 10). We extended this comparison to cell type-specific genes previously defined on transcriptomic reference sets derived from blood and bone marrow samples [10]. We observed the genes discriminating eosinophils, macrophages and neutrophils in our deconvolved asthma profiles predominantly overlapping with the respective genes defined on blood and bone-marrow data (see Additional file 1: Figure S20). However, the proportionate overlaps were smaller than with the gene set defined on lung eosinophils and in fact, a small number of discordances could be observed. Exemplarily, this will be further discussed in the following section.

## Discussion

Here, we present first whole-genome methylation data from sputum indicating that the methylation profile of sputum cells can be used to further the molecular characterization of chronic pulmonary inflammation in asthma and COPD. By performing omics deconvolution based on quadratic programming, taking sputum differential cell counts as input, we show that the analysis of mixed-cell sputum samples is strongly biased by the interindividual variation of cellular composition. In this context, we present data indicating a high potential of omics deconvolution to deliver results that are ultimately more closely relatable to pathophysiological regulation by making differential expression and methylation attributable to individual cell types.

Genomic methylation and gene expression represent distinct entities of cellular regulation [59]. Whilst promoter methylation has traditionally been connected to gene repression, recent advances have brought up a much more complex picture of epigenetic regulation and its influence on gene expression [60, 61]. Because epigenetic changes are thought to also reflect long-term alterations, they exhibit a strong potential for the profiling of chronic diseases linked to the environment, such as asthma and COPD [21, 62].

We set the scope of this study to be primarily methodology-oriented and used a limited number of biological replicates in our explorative analysis which imposes a limitation. As statistical analyses and particularly regression models rely on an adequate number of degrees of freedom, the presented results should be interpreted carefully and not be considered conclusive. However, though our study is underpowered to draw a detailed picture of the interaction of gene methylation and expression, we found methylation changes in genetic regions of interest.

In asthma macrophages, we observed differential methylation of the IL23A gene which could be seen in the context of macrophage polarization [63]. Furthermore, we found differential methylation related to the CCL24 (previously known as eotaxin-2) gene, which can potentially be attributed to macrophage differentiation, microbiota interaction [64] and eosinophil stimulation [65]. In asthma neutrophils, the IL5RA (interleukin 5 receptor alpha) gene was identified to be differentially methylated. Interestingly, though gene expression was estimated to be present only in eosinophils in our study (and IL5RA expression has traditionally been seen as eosinophil-specific), this had recently been described to be expressed by airway neutrophils in the context of treatment-refractory asthma in children [66].

In both asthma and COPD macrophages, our data indicated differential methylation of HLA genes related predominantly to MHC class II, but also class I molecules. We did not observe concordant expression changes; however, changes in methylation of HLA loci have been described in a variety of autoimmune diseases and states of immune dysregulation [67–70] and were linked to atopic asthma [71] in whole-blood profiling of children with atopy after rhinovirus-induced wheezing. In the latter study, differential methylation of SMAD3 (SMAD family member 3) was found to be particularly associated with asthma [71]. Congruently, in our analysis, we also observed the SMAD3 gene to lie within DMRs both in asthma and, interestingly, COPD macrophages (see Additional file 4).

A particular interesting case is the differential methylation and expression of CD8A in asthma that was counterintuitively attributed to macrophages. Since we were not able to reliably estimate lymphocyte profiles in our small data set and we did not use lymphocyte counts as independent predictors in the deconvolution, the possibility of this being a “contamination” by lymphocyte-specific expression and methylation arises. However, upon inspection of the differential cell counts for each sample, we did not observe collinearity between the macrophage and lymphocyte cell counts. In fact, several studies have described CD8A expression in (alveolar) macrophages before [72–75]. A more detailed discussion of potential sources of error in the deconvolution process is provided as supplementary information (Additional file 1).

Deconvolution of transcriptomic signatures further allowed to identify interesting candidates regarding cellular regulation: Our data indicated an upregulation of IL4R (interleukin 4 receptor) in asthma neutrophils which had been shown to play an important role in the regulation of neutrophil apoptosis [76]. Furthermore, CXCL2 was observed to be upregulated for which autocrine regulation of neutrophils had been demonstrated previously [77], offering potential to contribute to inflammation in asthma. In COPD, macrophages were found to upregulate CXCL9 expression which is known to be a macrophage-derived inflammatory cytokine and indicator of M1 differentiation [63], whereas upregulated expression of MMP13 could be attributed to imbalanced protease homeostasis [78, 79].

For now, the experimental gold standard to retrieve cell type-specific molecular data remains to be cellular separation by techniques such as gradient centrifugation [29, 30]. However, these methods do not always allow to purify more than one cell type simultaneously and their applicability on a large scale (e.g. in biobanking studies) may not be given due to infrastructural or financial limitations. In contrast, in silico deconvolution is suitable for application to data from conventionally processed whole-sputum samples. The required differential cell counts are frequently performed in cohort and clinical studies. Unfortunately, findings derived from deconvolved data, unless cellular separation had been performed in parallel, will often not be able to be validated in the same, mixed-cell sputum samples from which the omics data was generated (like in this study). However, quadratic programming has previously been found to deliver accurate deconvolution estimates [36]. Accordingly, we found an overall good agreement with the data published by Esnault et al. [29] for eosinophils. With higher biological replication in larger studies, the partial lack of estimation performance for some cell types (foremost lymphocytes) observed here is likely to resolve, allowing for an even more comprehensive gain of information by applying a deconvolution. In this context, the applicability of omics deconvolution to sputum data is not limited to methods based on manually performed sputum differential cell counts. Some approaches use cell type-specific transcriptomic reference profiles to infer the respective cellular quantities in mixed-cell samples and use these quantities for the deconvolution process subsequently [41]. An important pitfall that investigators should be aware of before employing such reference-based methods in sputum analyses becomes apparent from the comparison of our deconvolved data to cell type-specific gene sets defined on blood and bone marrow-derived data by Peters et al. [10]. Exemplarily, the gene GPR97 (G-protein coupled receptor 97, also known as adhesion G-protein coupled receptor G3, ADGRG3) was found to be selectively expressed in neutrophils in their analysis, whilst both the data by Esnault et al. and our deconvolved expression profiles indicated a predominant expression in eosinophils in the asthmatic lung environment. In fact, expression of GPR97 has been described for all granulocytes [80]. This does not contest the analysis of Peters et al. since they followed a completely different approach in analyzing the sputum transcriptome but is rather intended to illustrate the potential bias that can be introduced to estimating the cellular composition of sputum based on reference sets derived from other sources. If reference sets are used for the purpose of deconvolution, we recommend they should be created based on cells derived from the lung environment (sputum or BAL) in the respective disease state [81] which becomes particularly important since alveolar macrophages are considered to be developmentally distinct from monocyte (blood)-derived macrophages [82]. Otherwise, performing detailed differential cell counts as shown here is a viable alternative.

From a phenotypical perspective, a large variability of cellular composition within a given set of samples, whilst benefiting the fit of a regression model, potentially indicates that several disease entities are comprised (such as T2-low, T2-high, T2-ultra high etc. in asthma). The validity of estimates derived from deconvolution steps thereby directly depends on the accuracy of the preceding definition of sample groups. Therefore, the application of a regression-based deconvolution approach has to be critically evaluated in any experiment and might find complimentary use to deepen the molecular understanding after distinct pheno-/endotypes were separated (e.g. via the method established by Peters and colleagues [10]).

We further demonstrated that, should RNA degradation be of concern, e.g. due to suboptimal or long-term biobank storage, in silico correction can remove RNA integrity-associated bias from transcriptome data, thereby not only reducing the potential for the occurrence of false positives, but also increasing the overall sensitivity. In our data, a separate regression step performed well to remove degradation-related bias which is congruent with previous findings [83]. The cutoff we applied to select RNA samples supplied to microarray analysis was rather liberal (RIN > 3). Traditionally, cutoffs had been set to e.g. RIN > 5 [9, 84], at which it was demonstrated on cancer samples that the overall variance of gene expression is largely defined by interindividual differences and only to a much lesser extent by RNA integrity [84]. However, as overall expression differences in chronic inflammatory pulmonary disease may be more subtle than in cancer this cannot be easily assumed for sputum samples in asthma or COPD. Though several of the more strongly degraded samples in our study reached RIN values close to 5, they still clearly clustered separately from samples of higher RNA integrity. In fact, the principal component analysis of our transcriptome data showed that impaired RNA integrity has the potential of influencing the overall variance in transcription nearly as strongly as interindividual differences in asthma and COPD. Therefore, we suggest that the necessity of correction for RNA degradation should be evaluated even in data sets in which the sample quality had initially been judged suitable.

Thanks to optimized and streamlined purification workflows, parallel preparation of DNA and RNA from sputum samples is cost effective and efficient. With continuing innovation in the field of omics technologies and constantly growing affordability thereof, large-scale multi-omics analysis of sputum samples is close at hand. The application of the methods described here is by far not limited to sputum but can be expected to be successfully transferred to bronchoalveolar lavage and other respiratory samples to enhance biomarker discovery and pathophysiological understanding. Beyond microarray analysis, omics deconvolution and RNA integrity correction can further be expected to be of avail for sequencing-based methods as these can be similarly affected by RNA degradation and cell composition bias.

## Conclusions

Analysis of the sputum methylome can broaden the profiling and understanding of chronic pulmonary inflammation and adds important additional information to commonly performed transcription analyses. The necessity of in silico correction for RNA degradation should be evaluated in every sputum transcriptome dataset. Finally, with suitable deconvolution approaches such as the algorithm described here, pathophysiological and regulative changes in chronic inflammatory lung diseases can be substantially better explored wherever single-cell analysis or cell separation may not be feasible. We therefore strongly recommend the application of unbiased deconvolution methods as such to all future whole-sputum omics analyses in order to complement methods that have already been established.

## Supplementary information

**Additional file 1. **Supplementary Information, Figures and Tables.

**Additional file 2. ** Results of the differential expression analyses.

**Additional file 3.** Results of the differential methylation analyses on the CpG level.

**Additional file 4.** Results of the differential methylation analyses on the gene region level.

**Additional file 5.** Results of gene ontology enrichment analyses of the gene expression data.

**Additional file 6.** Results of KEGG pathway enrichment analyses of the gene expression data.

**Additional file 7.** Results of gene ontology enrichment analyses of the methylation data.

**Additional file 8.** Results of KEGG pathway enrichment analyses of the methylation data.

**Additional file 9.** Differentially expressed genes that were found to correspond to differentially methylated genomic regions.

**Additional file 10. **Genes whose expression was found to discriminate eosinophils, neutrophils and macrophages in asthma sputum.

## Data Availability

All transcriptome and methylome data have been deposited in NCBI’s Gene Expression Omnibus where they are publicly accessible through the GEO series accession numbers GSE148000 and GSE148004.

## Abbreviations

ACT: Asthma control test
BAL: Bronchoalveolar lavage
BH: Benjamini–Hochberg
COPD: Chronic obstructive pulmonary disease
cRNA: Complementary ribonucleic acid
DNA: Deoxyribonucleic acid
DEG: Differentially expressed gene
DMP: Differentially methylated position
DMR: Differentially methylated region
FACS: Fluorescence-activated cell sorting
FDR: False discovery rate
GO: Gene Ontology
GOLD: Global Initiative for Chronic Obstructive Lung Disease
HLA: Human Leukocyte Antigen
HOPE: Hepes-glutamic Acid Buffer-mediated Organic Solvent Protection Effect
ICS: Inhaled corticosteroids
KEGG: Kyoto Encyclopedia of Genes and Genomes
log_2_FC: Logarithmized (base 2) Fold Change
MHC: Major Histocompatibility Complex
PCR: Polymerase chain reaction
QP: Quadratic programming
RIN: RNA Integrity Number
RNA: Ribonucleic acid
SNP: Single nucleotide polymorphism
