# Supplementary material for: Exploration of the sputum methylome and omics deconvolution by quadratic programming in molecular profiling of asthma and COPD: the road to sputum omics 2.0
Source: Respir Res. 2020 Oct 19;21:274. doi: 10.1186/s12931-020-01544-4 (PMC7574293; doi:10.1186/s12931-020-01544-4)
Supplement: Supplementary file 1 — Additional file 1. Supplementary Information, Figures and Tables. [file 12931_2020_1544_MOESM1_ESM.pdf]

Exploration of the sputum methylome and omics deconvolution by quadratic programming in molecular profiling of asthma and COPD: the road to sputum omics 2.0

Espen E. Groth, Melanie Weber, Thomas Bahmer, Frauke Pedersen, Anne Kirsten, Daniela Börnigen, Klaus F. Rabe, Henrik Watz, Ole Ammerpohl, Torsten Goldmann

Additional File 1

Supplementary Information, Tables and Figures

Sputum induction and processing

Subjects underwent sputum induction by inhalation of nebulized saline in increasing concentrations from 0.9 to 3 % in repetitive inhalation/expectoration cycles (up to 4 per induction). Concomitant spirometry testing was performed to ensure subject safety during induction and saline concentration was not increased or induction of sputum was aborted if subjects experienced a decline in basal FEV1 of ≥ 10 or ≥ 20 %, respectively [1, 2].

Expectorated sputum was collected in petri dishes and assessed by microscopic evaluation. Dense plugs of sputum cells were manually separated from saliva and contaminants and incubated with dithiothreitol (Sputolysin®, Calbiochem/Merck, Darmstadt, Germany). After addition of phosphate-buffered saline (PBS), sputum cell suspensions were filtered through nylon mesh (53 µm pore size) and pelleted by centrifugation. Following removal of supernatants, cell pellets were resuspended in PBS and aliquots for subsequent cell counting with a hemocytometer were taken, followed by preparation of cell slides. Before further preservation and storage, remaining sputum cells were pelleted by centrifugation.

Sputum differential cell counts

No substantial differences in the distribution of cellular proportions could be observed between the samples submitted to methylation and transcription profiling (see Supplementary Tables S1 and S2 as well as Supplementary Figure S1).

Table S1: Differential cell count of sputum samples supplied to methylation microarray analysis

	AM	NG	EO	LY	MO	CC	SC
**Asthma**<br>n = 9	27.9 ± 21.9<br>(6.3/60.4)	54.7 ± 24.4<br>(14.1/84.8)	12.9 ± 24.5<br>(1.5/77.0)	0.7 ± 0.5<br>(0.1/1.6)	0.1 ± 0.1<br>(0.0/0.3)	1.6 ± 1.0<br>(0.5/3.3)	2.1 ± 3.4<br>(0.3/10.8)
**COPD**<br>n = 10	9.0 ± 5.9<br>(1.1/21.1)	88.9 ± 6.6<br>(76.6/98.1)	1.0 ± 1.2<br>(0.0/4.0)	0.2 ± 0.3<br>(0.0/0.8)	0.0	0.4 ± 0.4<br>(0.0/1.4)	0.6 ± 0.5<br>(0.0/1.8)
**Controls**<br>n = 7	47.8 ± 23.3<br>(16.3/81.3)	43.3 ± 24.4<br>(6.5/76.1)	0.3 ± 0.4<br>(0.0/1.1)	2.1 ± 2.6<br>(0.0/7.6)	0.3 ± 0.3<br>(0.0/0.9)	1.8 ± 0.7<br>(0.9/2.6)	4.4 ± 3.5<br>(0.4/10.4)

Cell proportions are reported as mean percentage ± SD (min/max).
AM: alveolar macrophages. NG: neutrophil granulocytes. EO: eosinophils. LY: lymphocytes. MO: monocytes. CC: ciliated cells (respiratory epithelium). SC: squamous cells.

Table S2: Differential cell count of sputum samples supplied to gene expression microarray analysis.

	AM	NG	EO	LY	MO	CC	SC
**Asthma**<br>n = 9	27.9 ± 21.9<br>(6.3/60.4)	54.7 ± 24.4<br>(14.1/84.8)	12.9 ± 24.5<br>(1.5/77.0)	0.7 ± 0.5<br>(0.1/1.6)	0.1 ± 0.1<br>(0.0/0.3)	1.6 ± 1.0<br>(0.5/3.3)	2.1 ± 3.4<br>(0.3/10.8)
**COPD**<br>n = 7	9.9 ± 6.4<br>(2.8/21.1)	88.4 ± 6.7<br>(76.6/96.1)	0.6 ± 0.6<br>(0.0/1.8)	0.2 ± 0.3<br>(0.0/0.8)	0.0	0.4 ± 0.5<br>(0.0/1.4)	0.6 ± 0.6<br>(0.0/1.8)
**Controls**<br>n = 9	52.8 ± 26.3<br>(16.3/81.3)	40.1 ± 26.6<br>(6.5/76.1)	0.3 ± 0.4<br>(0.0/1.1)	2.1 ± 2.2<br>(0.0/7.6)	0.2 ± 0.3<br>(0.0/0.9)	1.6 ± 0.9<br>(0.4/2.6)	3.0 ± 3.2<br>(0.4/10.4)

Cell proportions are reported as mean percentage ± SD (min/max).
AM: alveolar macrophages. NG: neutrophil granulocytes. EO: eosinophils. LY: lymphocytes. MO: monocytes. CC: ciliated cells (respiratory epithelium). SC: squamous cells.

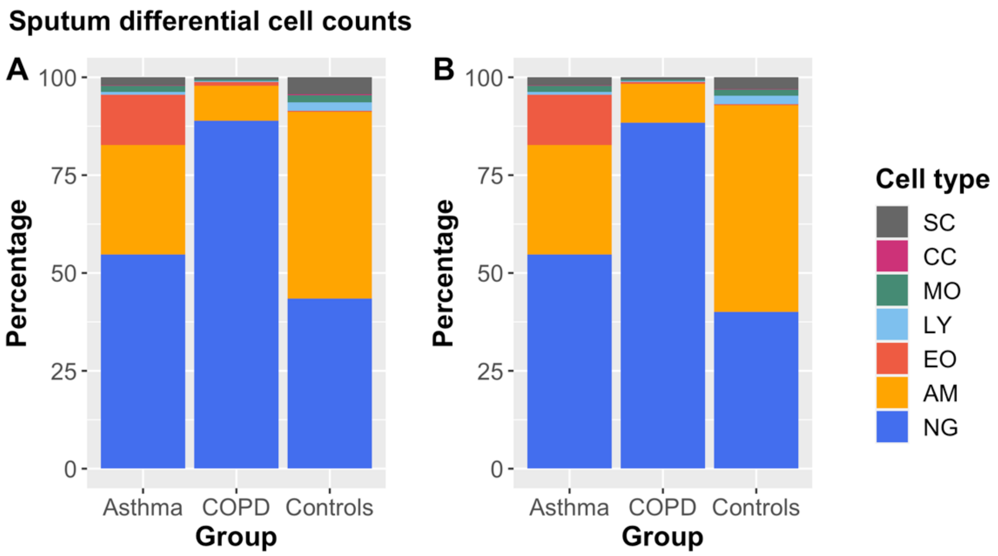

Figure S1: Mean cellular composition of sputum samples, shown separately for samples supplied to methylation profiling (A) and gene expression profiling (B).
AM: alveolar macrophages. NG: neutrophil granulocytes. EO: eosinophils. LY: lymphocytes. MO: monocytes. CC: ciliated cells (respiratory epithelium). SC: squamous cells.

R/Bioconductor software packages

Table S3: Software packages utilized for statistical analysis and data display.

Name	Version	Reference
R	3.6.1	[3]
devtools	2.2.2	[4]
Bioconductor	3.10.1	[5]
limma	3.42.2	[6]
minfi	1.32.0	[7]
IlluminaHumanMethylation450kanno.ilmn12.hg19	0.6.0	[8]
IlluminaHumanMethylation450kmanifest	0.4.0	[9]
RColorBrewer	1.1-2	[10]
matrixStats	0.56.0	[11]
DMRcate	2.0.7	[12]
DMRcatedata	2.2.1	[13]
quadprog	1.5-8	[14]
gtools	3.8.1	[15]
BSDA	1.2.1	[16]
plyr	1.8.6	[17]
clusterProfiler	3.14.3	[18]
org.Hs.eg.db	3.10.0	[19]
msigdbr	7.0.1	[20]
tidyr	1.0.2	[21]
ggplot2	3.3.0	[22]
cowplot	1.0.0	[23]
VennDiagram	1.6.20	[24]
EDec	0.9	[25]

DMR identification with *DMRcate*

All workflow steps were calculated based on methylation beta values at standard kernel settings (bandwidth lambda = 1000, scaling factor C = 2, minimum no. of consecutive CpGs = 2).
From the deconvolved data, we supplied the measures of significance of differential expression/methylation, together with the corresponding deconvolution estimates, to internal functions of the package.

Enrichment analysis with *clusterProfiler*

Gene symbols were mapped to Entrez IDs based on information of the org.Hs.eg.db annotation package.

Correction for RNA degradation

From the framework of available omics technologies, transcriptomics has by far been the one most frequently applied to sputum samples [26, 27]. However, the process of isolating high-quality nucleic acids from sputum is not trivial. Preparation of suitable samples from raw, freshly expectorated sputum is intensive and requires purifying procedures to remove saliva and break up mucin bonds by incubation with reducing chemicals. In light of the general instability of ribonucleic acid (RNA), being targeted by degrading enzymes (RNases) in the environment, challenges for handling and storage of sputum samples become obvious [28]. Methods that focus on high-throughput gene expression profiling (such as transcription microarray analysis or RNA sequencing) are optimized to work with very small amounts of input RNA and usually employ RNA amplification steps in their workflows. In the case of messenger RNA (mRNA) profiling, these are commonly based on poly-thymine (poly-T) primers that bind to poly-adenin sequences (poly-A tails), allowing for selective human mRNA amplification [29]. This has potential to introduce substantial bias to downstream analyses in light of RNA degradation. In case of the widely spread microarray platforms, transcripts may not be detected if their corresponding array probes map to distant transcript regions that had already been cleaved from the poly-A-adjacent fragment. Previous studies on this issue have shown that the exclusion of array probes from downstream analyses, based on the position in the corresponding transcript to which they map, does not necessarily remove RNA quality effects from transcriptome data. Instead, RNA degradation in biological materials is considered to be a dynamic process that not solely depends on the length of RNA molecules (as, in contrast, it can be assumed for purified RNA samples) [30, 31]. Though sputum processing and storage protocols have been optimized to ensure retrieval of high-quality RNA, degradation remains a challenge to consider in any large-scale prospective biobank study in which sample collection and analysis may take place years apart. As long as the overall variation of RNA quality can be assumed to be constant across sample groups, the effects specified above may primarily impair the sensitivity of comparative transcriptome studies. When RNA quality is found to be distributed unequally across sample groups, however, degradation may in fact lead to false-positive findings. In the context of inter-array quantile normalization, a rank-based normalization algorithm frequently applied to gene expression microarrays [32, 33], transcripts can in fact be rendered to both higher and lower expression levels upon RNA degradation [31]. Some *in silico* correction methods [31] have been suggested to overcome these challenges but have not been evaluated in the context of sputum analysis yet.

We evaluated two correction procedures for RNA degradation: First, we calculated the correlation (Pearson's product moment correlation coefficient r together with the associated two-sided p value of the correlation test) between expression and RIN (RNA Integrity Number) values across samples for each array probe. As expected in the context of rank-based normalization, gene expression was both positively (52.7 % of transcripts) and negatively (47.3 %) correlated with RIN in our data. In total, 43.2 % of transcripts were correlated at $p < 0.1$ and 36.1 % at $p < 0.05$ (see Figure S2). Probes who showed a correlation of $r \geq 0.3$ (to be seen as medium association, based on the definition of Cohen [34]) at $p < 0.1$ were removed from the data set, followingly referred to as correlation filtering.

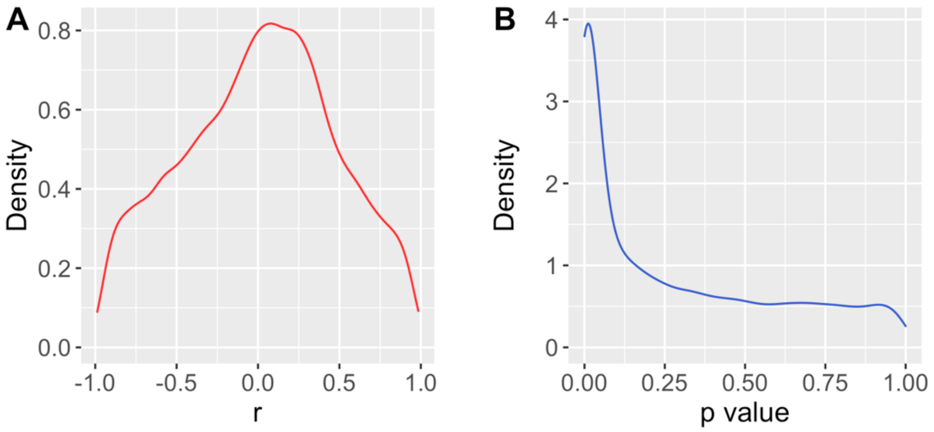

Figure S2: Correlation between measured gene expression and RNA integrity (RIN, RNA Integrity Number). Distribution of r (Pearson correlation coefficient, A) and associated p values (B).

Second, to correct for RNA degradation by linear least-squares regression, we applied a simple model of the form

$$y_i = \beta_0 + \beta_1 x_i + \varepsilon_i \quad \text{with} \quad i = 1, \dots, n \qquad (1)$$

where n represents the number of samples supplied to the regression. For every sample i, x_i denotes the single scalar predictor variable, y_i the single scalar response variable and ε_i the stochastic component. β_0 and β_1 denote the regression parameters (β_0 can be referred to as intercept). In our case, we defined the RNA integrity (represented by the RNA integrity number, RIN) as predictor and the measured (log_2) gene expression value as response variable. Consequently, β_1 represents the estimated measure of association between RNA integrity and expression, β_0 (intercept) the approximated integrity-independent measure of expression with an estimate of variation across samples retained in ε. Reversely calculating $\{y_i\}$ based on the estimates β_0, β_1 and $\{\varepsilon_i\}$ with $\{x_i\}$ set to the maximum RIN present in our study (9.1), we were able to retrieve expression values corrected for RIN-related effects (see also Figure S3). This was implemented with built-in functions of R (*stats* R core package).

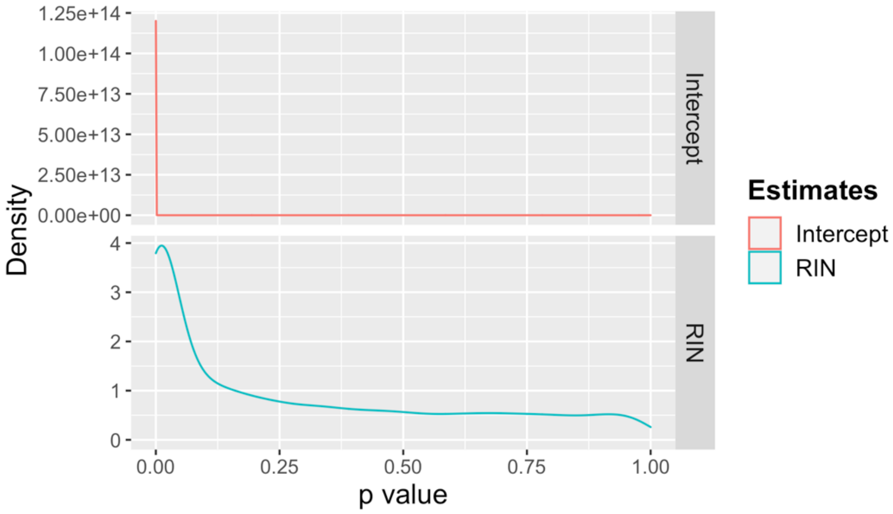

Figure S3: Applying linear regression for RNA integrity (RIN) correction: distribution of p values associated with regression parameters.

Before applying the correlation filtering or linear regression steps to the expression dataset, we took into account that the COPD group contained a higher proportion of HOPE-preserved samples with therefore more intensely degraded RNA than the asthma and control groups (see Table 3 in main manuscript). Therefore, correlation and regression characteristics were determined based on the asthma and control samples and then subsequently applied to the COPD samples in order to control for a possible skewing of correlation and regression parameters by COPD-specific expression patterns [31].

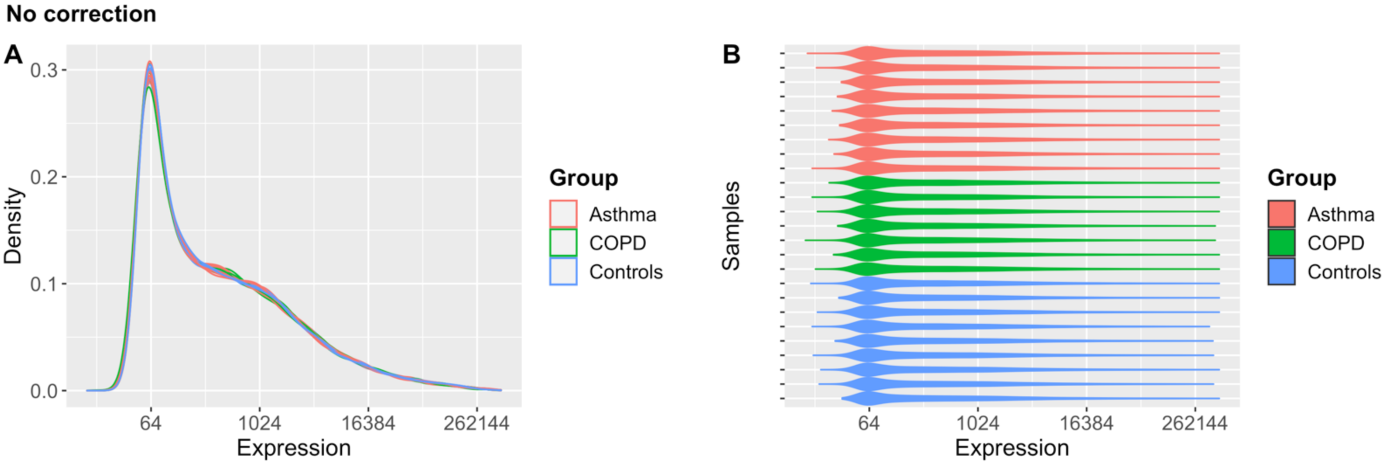

Figure S4: Distribution of gene expression values before correction for RNA integrity: density plot (A) and bean plot (B).

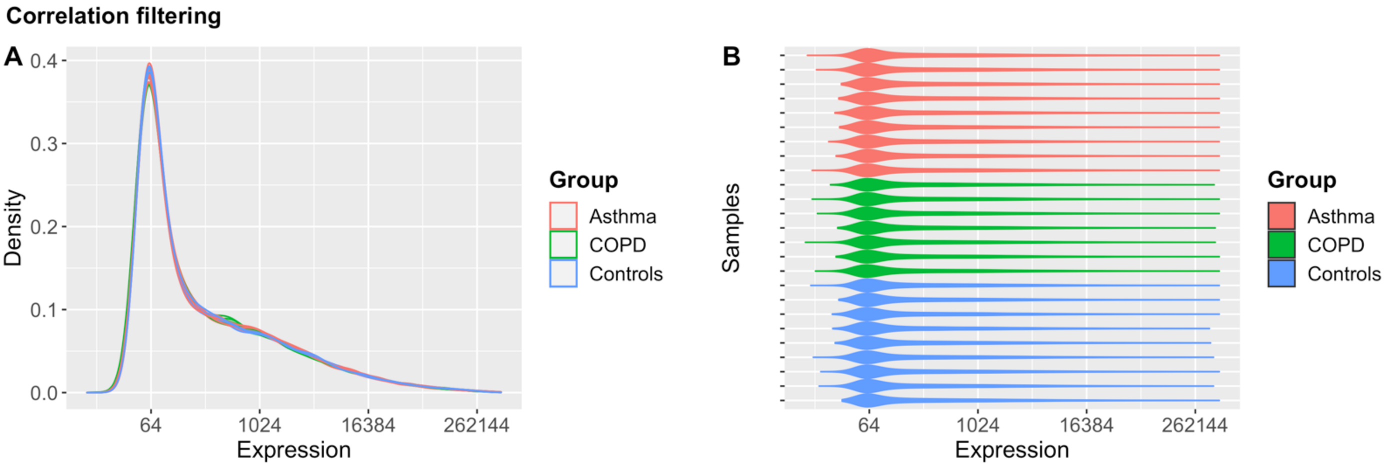

Figure S5: Distribution of gene expression values after correlation filtering: density plot (A) and bean plot (B).

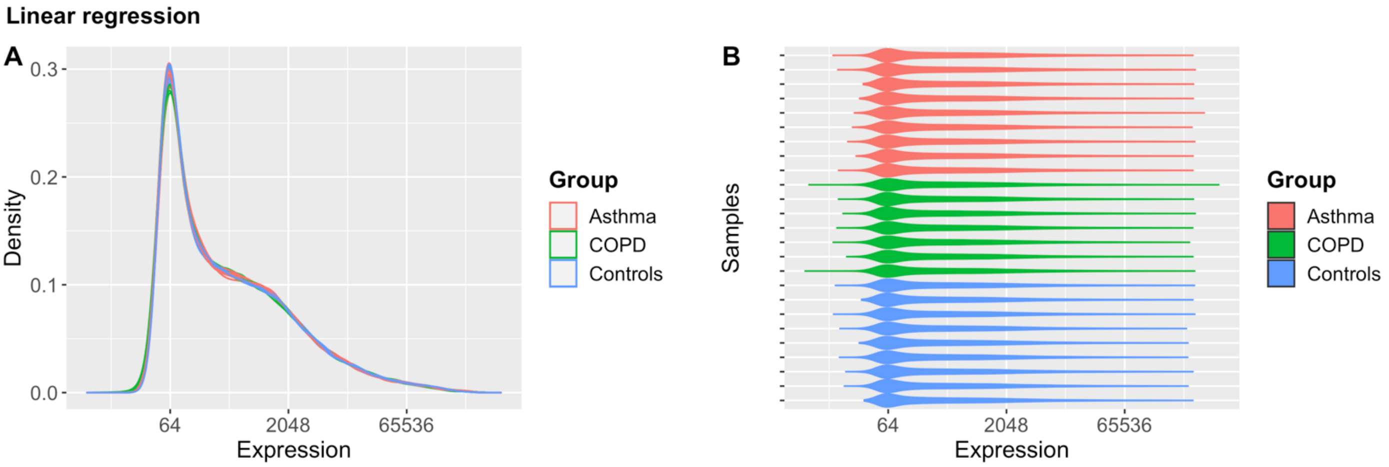

Figure S6: Distribution of gene expression values after correction for RNA integrity by linear regression: density plot (A) and bean plot (B).

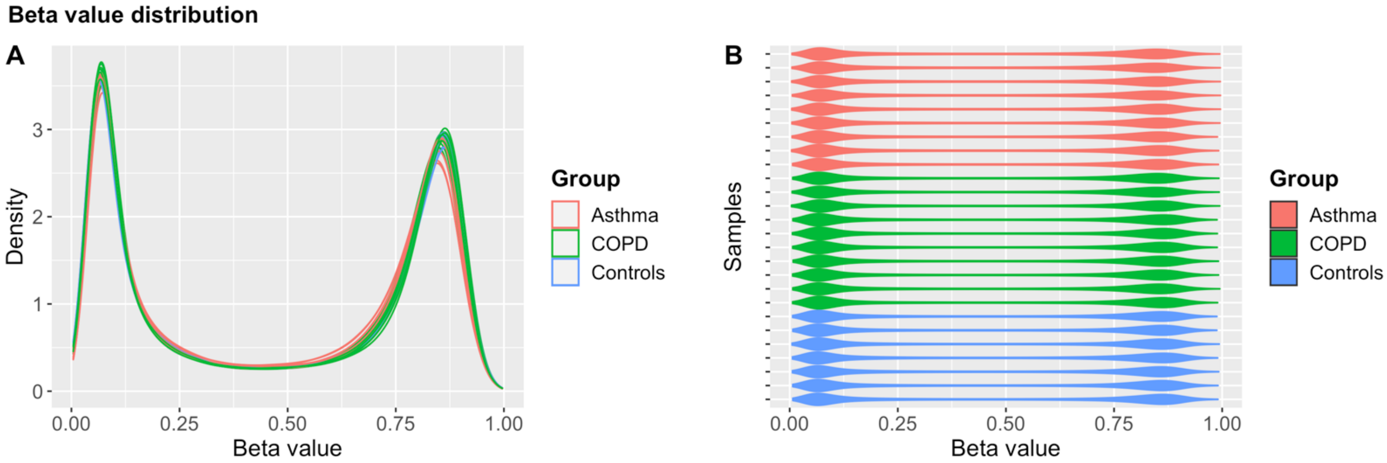

Figure S7: Distribution of beta values in the methylome data: density plot (A) and bean plot (B).

Estimation of cell type-specific gene expression and methylation (deconvolution)

Based on the general assumption that both the methylation level of any CpG and the expression level of any transcript measured in a mixed-cell sample (such as sputum) result from the linear combination of the methylation/expression levels of all analyzed cells (as it were the "mean" of expression/methylation of all cells), and further assuming that methylation/gene expression across the cells of a particular cell type can be seen as homogeneous, inferring cell type-specific omics patterns from mixed-cell measurements can be reduced to a multiple (linear) regression problem in case the cellular proportions are known:

$$y = X\beta + \varepsilon \tag{2}$$

Here, $X = \{x_{ij}\}$ represents the matrix of predictor variables where column j relates to the proportionate quantity of cell type $j = 1, \ldots, c$ across samples $i = 1, \ldots, n$. $y = \{y_i\}$ denotes the vector of response variables (measured methylation/expression), $\beta = \{\beta_j\}$ the vector of regression parameters (estimated cell type-specific methylation/gene expression) and $\varepsilon = \{\varepsilon_i\}$ the vector of stochastic components (residuals). Fitting the above model by the standard least-squares method, however, disregards that methylation and expression values in the biological context represent bounded variables: there exists no methylation below 0 or over 100 % and, likewise, there exists no negative expression. Therefore, during the regression process, an optimal solution has to be found which assigns to all cell types expression/methylation levels that are biologically possible. As a solution, we performed regression by quadratic programming, allowing us to specify (biological) constraints (C in (3)) under which the regression parameters were estimated. This approach had previously been successfully applied to methylation and gene expression data [35, 36]. Programmatically, we implemented the dual method of Goldfarb and Idnani [37] (via the *quadprog* package) to solve the problem

$$\arg min_{\beta} \parallel y - X\beta \parallel^2 \quad \text{with} \quad \beta \in C \tag{3}$$

The assumption of linear combinability can be seen to hold true for methylation reported on the beta value scale (where values approximately correspond to proportionate methylation). Since the commonly applied $\log_2$-transformation of expression values does not allow for linear combination, expression values had to be analyzed on the linear (instead of $\log_2$-transformed) scale.
Estimation was performed for each sample group (asthma, COPD and controls) separately. Methylation estimates were constrained to $C = [0, 1]$ and expression estimates to the dynamic range of the array (defined as respective minimum and maximum background corrected feature intensities after quantile normalization).
Following a standard approach in regression analysis as previously implemented by Onuchic et al. [35], we estimated the standard error s_j of each of the regression estimates (methylation/gene expression level in each cell type j) in the same way as for a multiple linear regression problem by

$$s_j = \sqrt{[MSE\ (X^t X)^{-1}]_{j,j}} \tag{4}$$

where *MSE* denotes the mean squared error, calculated by

$$MSE = \frac{\sum_{i=1}^{n} {\varepsilon_i}^2}{n - c} \qquad (5)$$

In (5), the mean squared error (MSE) is considered to be an unbiased estimator of the true (unknown) mean squared error by dividing by the degrees of freedom [38].

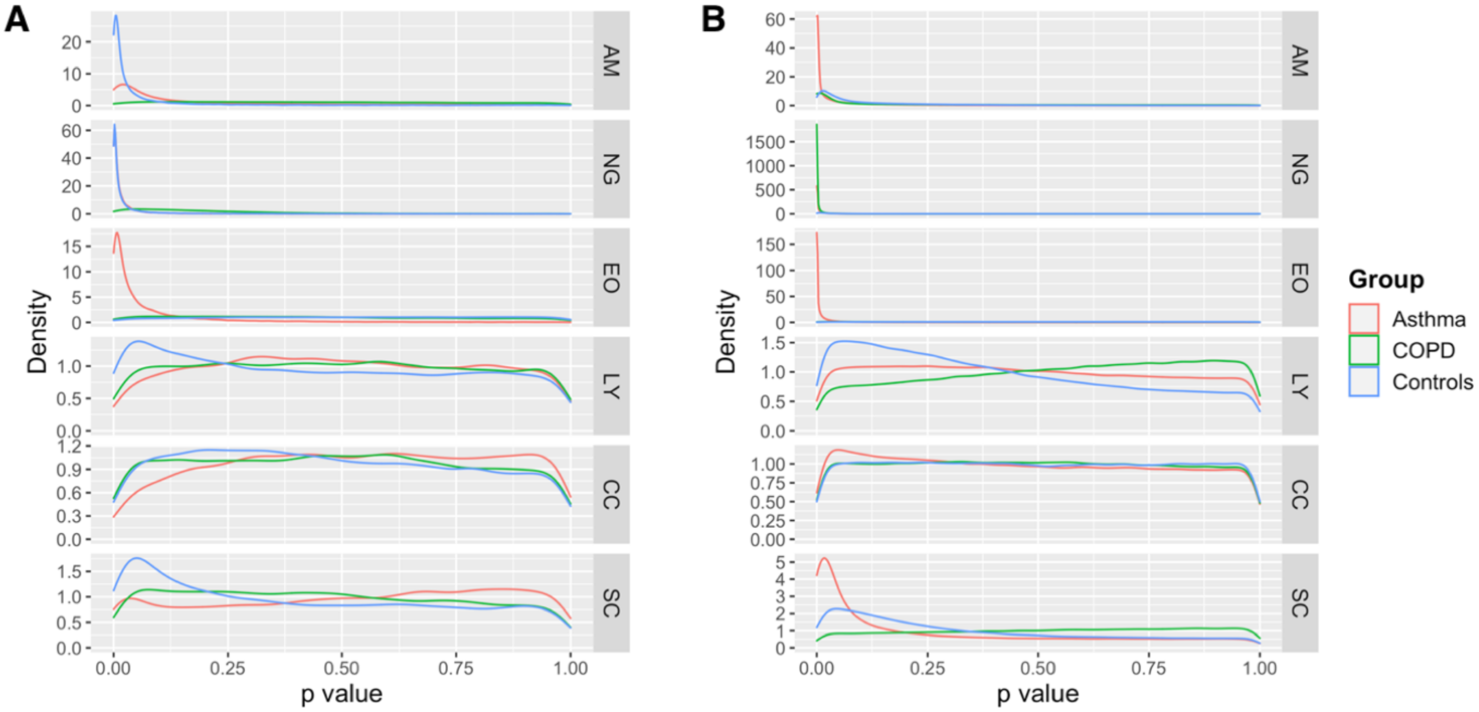

Figure S8: Distribution of the regression p values associated with fitting multiple linear models to the gene expression (A) and methylation data (B).
AM: alveolar macrophages. NG: neutrophil granulocytes. EO: eosinophils. LY: lymphocytes. CC: ciliated cells (respiratory epithelium). SC: squamous cells.

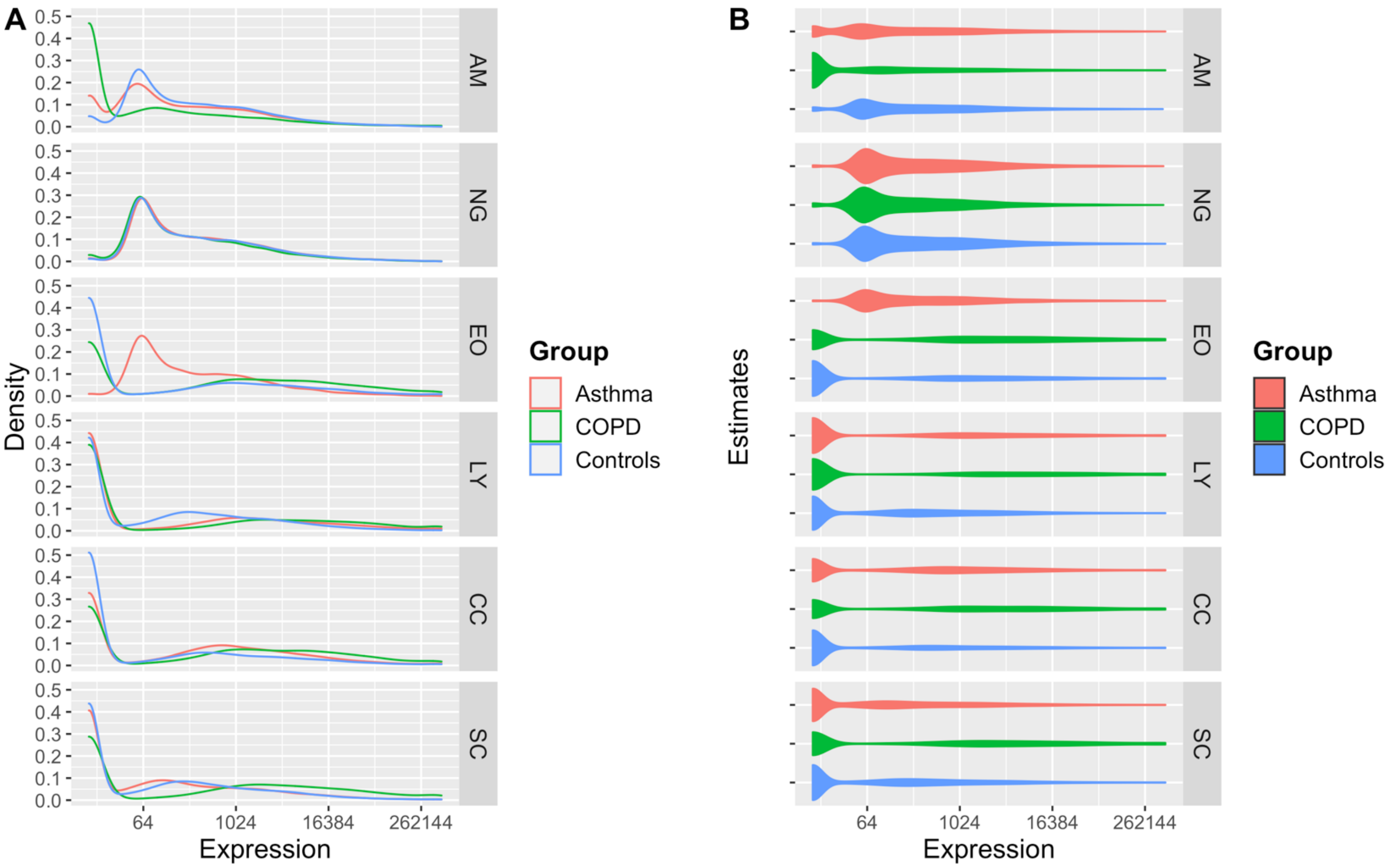

Figure S9: Distribution of the cell type-specific gene expression estimates after deconvolution: density plot (A) and bean plot (B).
AM: alveolar macrophages. NG: neutrophil granulocytes. EO: eosinophils. LY: lymphocytes. CC: ciliated cells (respiratory epithelium). SC: squamous cells.

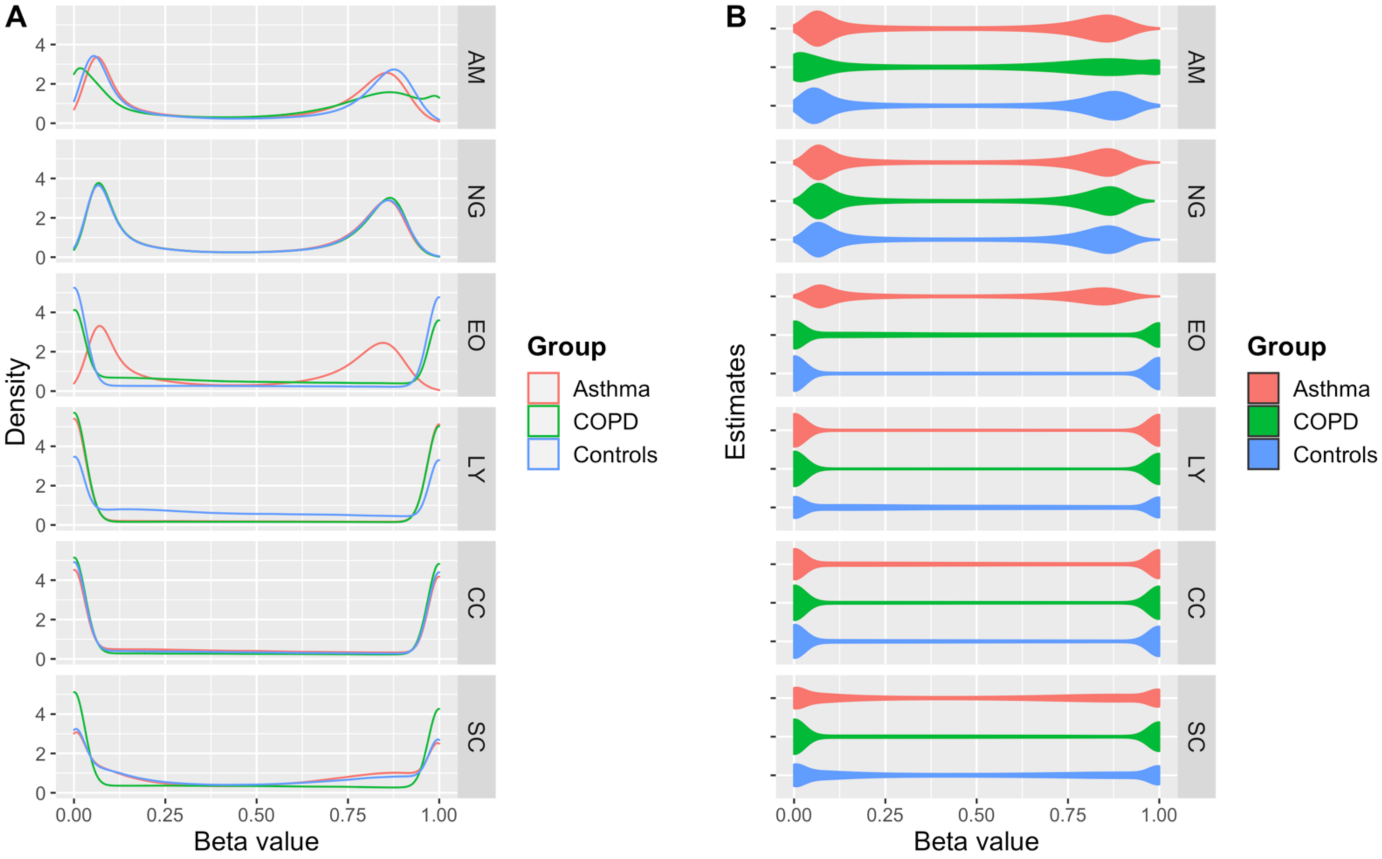

Figure S10: Distribution of the cell type-specific methylation beta value estimates after deconvolution: density plot (A) and bean plot (B).
AM: alveolar macrophages. NG: neutrophil granulocytes. EO: eosinophils. LY: lymphocytes. CC: ciliated cells (respiratory epithelium). SC: squamous cells.

Table S4: Percentiles of the regression p value distributions associated with fitting multiple linear models to the gene expression data.

Group	Cell	Percentiles 5%	10%	25%	50%	75%	95%	98%	99%	% sig at p < 0.05	p < 0.001
**Asthma**	**AM**	0.00	0.01	0.02	0.07	0.26	0.79	0.92	0.96	41.2	1.6
	**NG**	0.00	0.00	0.00	0.01	0.02	0.13	0.33	0.56	87.3	13.1
	**EO**	0.00	0.00	0.01	0.03	0.09	0.43	0.69	0.84	62.4	5.1
	**LY**	0.07	0.12	0.27	0.50	0.74	0.95	0.98	0.99	3.8	0.1
	**CC**	0.08	0.15	0.30	0.54	0.77	0.95	0.98	0.99	2.8	0.1
	**SC**	0.03	0.08	0.27	0.54	0.78	0.96	0.98	0.99	7.4	0.3
**COPD**	**AM**	0.04	0.09	0.22	0.45	0.70	0.94	0.98	0.99	5.7	0.1
	**NG**	0.01	0.03	0.07	0.16	0.28	0.61	0.82	0.91	17.0	0.4
	**EO**	0.04	0.08	0.21	0.43	0.70	0.94	0.98	0.99	6.3	0.1
	**LY**	0.05	0.10	0.25	0.49	0.74	0.95	0.98	0.99	4.9	0.1
	**CC**	0.05	0.10	0.24	0.49	0.72	0.95	0.98	0.99	5.2	0.1
	**SC**	0.04	0.08	0.22	0.45	0.71	0.94	0.98	0.99	6.0	0.2
**Controls**	**AM**	0.00	0.00	0.00	0.02	0.06	0.57	0.84	0.92	71.3	7.6
	**NG**	0.00	0.00	0.00	0.01	0.03	0.20	0.45	0.65	84.4	14.1
	**EO**	0.06	0.12	0.28	0.53	0.77	0.95	0.98	0.99	4.1	0.1
	**LY**	0.02	0.06	0.18	0.43	0.72	0.94	0.98	0.99	9.0	0.2
	**CC**	0.05	0.10	0.23	0.46	0.71	0.94	0.98	0.99	4.8	0.1
	**SC**	0.02	0.04	0.14	0.38	0.68	0.94	0.97	0.99	11.7	0.3

AM: alveolar macrophages. NG: neutrophil granulocytes. EO: eosinophils. LY: lymphocytes. CC: ciliated cells (respiratory epithelium). SC: squamous cells.

Table S5: Percentiles of the regression p value distributions associated with fitting multiple linear models to the methylation data.

Group	Cell	Percentiles 5%	10%	25%	50%	75%	95%	98%	99%	% sig at p < 0.05	p < 0.001
**Asthma**	**AM**	0.00	0.00	0.00	0.01	0.05	0.34	0.59	0.75	74.5	33.3
	**NG**	0.00	0.00	0.00	0.00	0.00	0.04	0.08	0.13	96.0	59.4
	**EO**	0.00	0.00	0.00	0.00	0.01	0.12	0.27	0.41	88.9	48.2
	**LY**	0.05	0.09	0.23	0.47	0.72	0.94	0.98	0.99	5.2	0.1
	**CC**	0.04	0.08	0.22	0.47	0.73	0.95	0.98	0.99	6.1	0.1
	**SC**	0.00	0.01	0.03	0.16	0.53	0.90	0.96	0.98	32.3	2.0
**COPD**	**AM**	0.00	0.00	0.00	0.06	0.39	0.87	0.95	0.97	47.7	13.1
	**NG**	0.00	0.00	0.00	0.00	0.00	0.03	0.07	0.12	97.2	68.5
	**EO**	0.03	0.07	0.20	0.45	0.72	0.94	0.98	0.99	7.9	0.2
	**LY**	0.07	0.13	0.31	0.56	0.79	0.96	0.98	0.99	3.6	0.1
	**CC**	0.05	0.10	0.25	0.49	0.74	0.95	0.98	0.99	5.2	0.1
	**SC**	0.06	0.12	0.29	0.54	0.78	0.96	0.98	0.99	4.2	0.1
**Controls**	**AM**	0.00	0.01	0.02	0.06	0.19	0.51	0.71	0.83	44.9	1.2
	**NG**	0.00	0.00	0.01	0.03	0.09	0.26	0.40	0.51	62.4	2.5
	**EO**	0.05	0.11	0.26	0.52	0.76	0.95	0.98	0.99	4.7	0.1
	**LY**	0.03	0.07	0.17	0.37	0.64	0.92	0.97	0.98	7.7	0.2
	**CC**	0.05	0.10	0.25	0.50	0.75	0.95	0.98	0.99	5.0	0.1
	**SC**	0.02	0.04	0.11	0.28	0.57	0.91	0.96	0.98	11.8	0.2

AM: alveolar macrophages. NG: neutrophil granulocytes. EO: eosinophils. LY: lymphocytes. CC: ciliated cells (respiratory epithelium). SC: squamous cells.

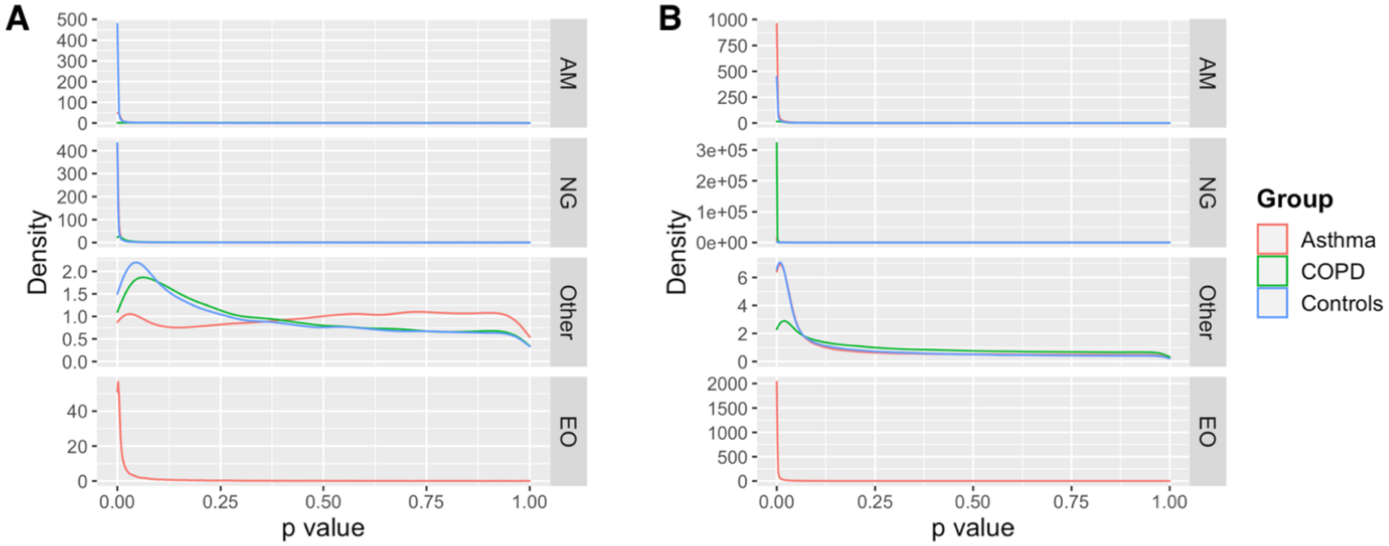

Figure S11: Distribution of the regression p values associated with fitting multiple linear models to the gene expression (A) and methylation data (B) after summarization of cell types with low prevalence.
AM: alveolar macrophages. NG: neutrophil granulocytes. EO: eosinophils. Other: sum of residual cell types (weighed intercept).

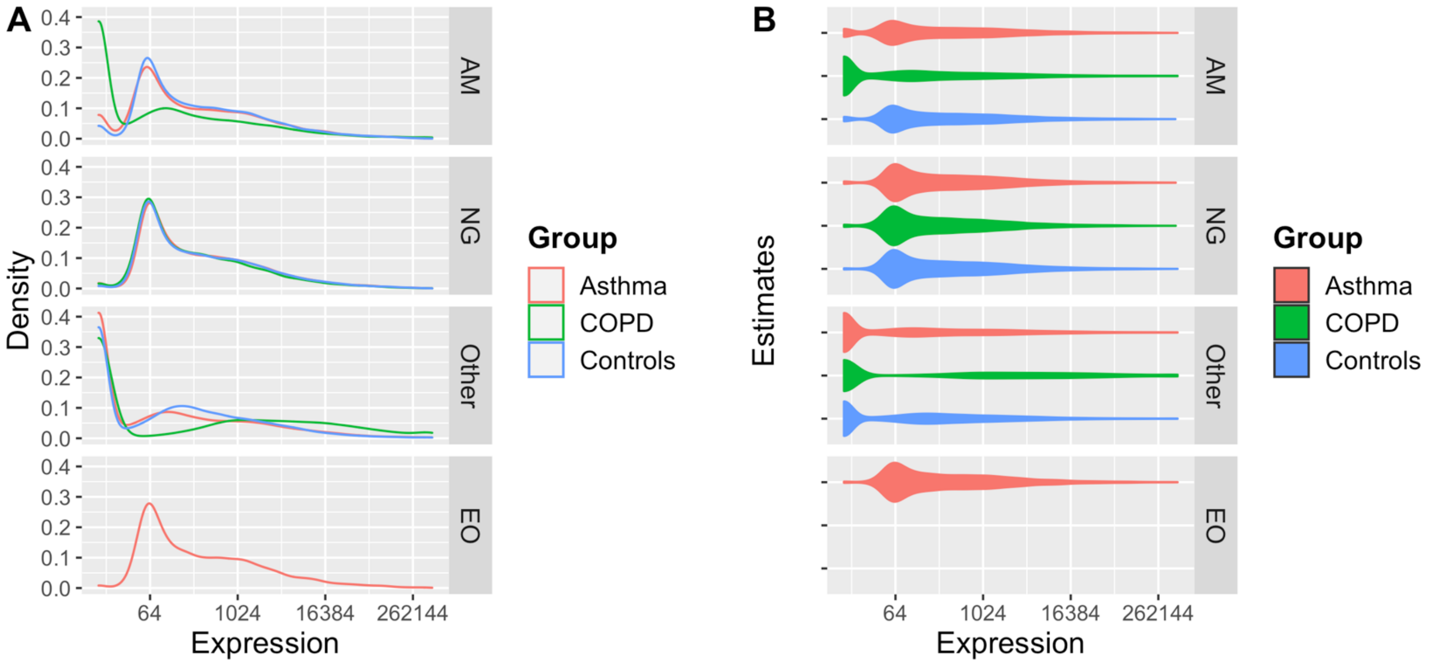

Figure S12: Distribution of the cell type-specific gene expression estimates after deconvolution with the reduced set of cell types: density plot (A) and bean plot (B).
AM: alveolar macrophages. NG: neutrophil granulocytes. EO: eosinophils. Other: sum of residual cell types (weighed intercept).

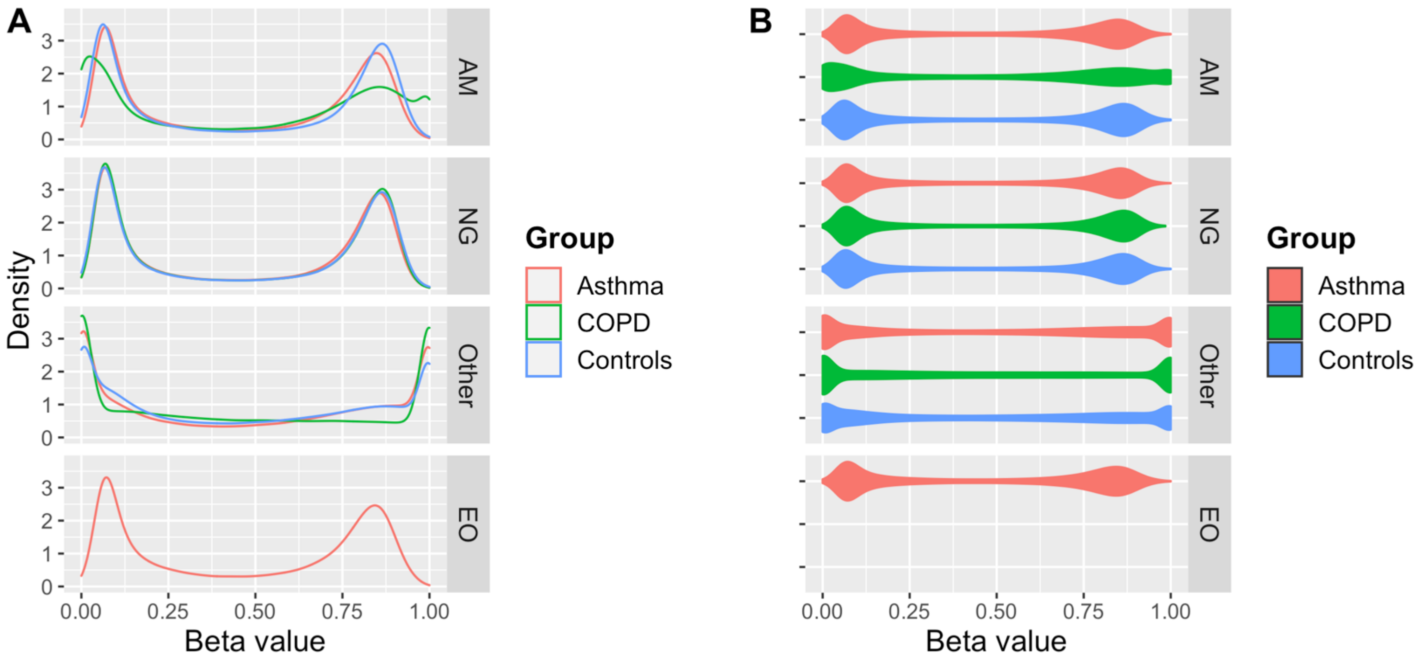

Figure S13: Distribution of the cell type-specific methylation beta value estimates after deconvolution with the reduced set of cell types: density plot (A) and bean plot (B).
AM: alveolar macrophages. NG: neutrophil granulocytes. EO: eosinophils. Other: sum of residual cell types (weighed intercept).

Table S6: Percentiles of the regression p value distributions associated with fitting multiple linear models to the gene expression data after summarization of cell types with low prevalence.

		Percentiles								% sig at	
**Group**	**Cell**	5%	10%	25%	50%	75%	95%	98%	99%	p < 0.05	p < 0.001
**Asthma**	**AM**	0.00	0.00	0.00	0.00	0.04	0.60	0.83	0.92	76.2	27.9
	**NG**	0.00	0.00	0.00	0.00	0.00	0.07	0.34	0.56	93.6	52.5
	**Other**	0.02	0.07	0.26	0.53	0.77	0.95	0.98	0.99	8.5	0.6
	**EO**	0.00	0.00	0.00	0.01	0.04	0.32	0.59	0.77	79.2	24.0
**COPD**	**AM**	0.03	0.06	0.18	0.40	0.68	0.93	0.97	0.99	8.2	0.2
	**NG**	0.00	0.00	0.00	0.02	0.07	0.46	0.75	0.87	70.3	10.1
	**Other**	0.02	0.04	0.13	0.33	0.63	0.93	0.97	0.99	11.1	0.2
**Controls**	**AM**	0.00	0.00	0.00	0.00	0.00	0.23	0.59	0.79	88.6	63.5
	**NG**	0.00	0.00	0.00	0.00	0.01	0.15	0.41	0.57	90.6	56.2
	**Other**	0.01	0.03	0.10	0.31	0.62	0.93	0.97	0.98	15.3	0.4

AM: alveolar macrophages. NG: neutrophil granulocytes. EO: eosinophils. Other: Sum of residual cell types (weighed intercept).

Table S7: Percentiles of the regression p value distributions associated with fitting multiple linear models to the methylation data after summarization of cell types with low prevalence.

		Percentiles								% sig at	
**Group**	**Cell**	5%	10%	25%	50%	75%	95%	98%	99%	p < 0.05	p < 0.001
**Asthma**	**AM**	0.00	0.00	0.00	0.00	0.00	0.07	0.18	0.31	93.8	66.0
	**NG**	0.00	0.00	0.00	0.00	0.00	0.01	0.03	0.06	98.7	81.4
	**Other**	0.00	0.00	0.01	0.10	0.49	0.90	0.96	0.98	41.9	8.2
	**EO**	0.00	0.00	0.00	0.00	0.00	0.05	0.15	0.27	95.0	70.1
**COPD**	**AM**	0.00	0.00	0.00	0.02	0.26	0.82	0.92	0.96	56.7	35.0
	**NG**	0.00	0.00	0.00	0.00	0.00	0.00	0.01	0.02	99.6	93.6
	**Other**	0.01	0.02	0.09	0.31	0.63	0.92	0.97	0.98	18.0	1.6
**Controls**	**AM**	0.00	0.00	0.00	0.00	0.01	0.11	0.29	0.49	91.3	58.8
	**NG**	0.00	0.00	0.00	0.00	0.00	0.05	0.13	0.25	95.4	67.0
	**Other**	0.00	0.00	0.01	0.10	0.44	0.88	0.95	0.98	41.5	8.5

AM: alveolar macrophages. NG: neutrophil granulocytes. EO: eosinophils. Other: Sum of residual cell types (weighed intercept).

When implementing a regression model-based deconvolution approach several points have to be critically considered. The model's performance is not only dependent on the achieved number of degrees of freedom, but also directly dependent on the quality and accuracy of the input data. Sputum differential cell counts should therefore be prepared with the highest resolution possible. However, cell types that only represent a small proportion of the cell set or that cannot be identified via a conventional differential cell count (such as specific subsets of lymphocytes) won't be able to be estimated via the regression. The overall performance of the model in our data shows that this holds true: estimates were anticipated to be most reliable for those cell types that were most prevalent whilst exhibiting variability within the respective sample group. Consequently, in our data, this led to the estimation of expression and methylation in eosinophils only performing well in asthma samples. In larger-scale studies with well-defined phenotypes, however, it may well be possible to compare transcription/methylation profiles of eosinophils, e.g. between asthma subgroups.

One of the key assumptions on which the here described models rely is linearity (linear combinability) of the data. For methylation measured by beta values from 0 to 1 this can be seen as holding true, though, needless to say, beta values themselves represent an estimate of the overall methylation level in a given set of cells (there exists no “50 % CpG methylation” in a single DNA strand as methylation is either present or absent for a single CpG site). In case of expression data, specifically from microarray experiments, however, a restricted dynamic range and the effect of fold change compression [39], are complications to the linearity assumption. Moreover, not every cell or cell type necessarily contributes the overall same amount of RNA molecules to the extract from mixed-cell samples whereas the amount of contributed DNA (copies of the genome) is constant. As RNA is generally more unstable than DNA, the RNA amounts contributed by cells that are seen as contaminants in sputum (such as respiratory epithelium or squamous cells), which, due to their loss of original cellular integrity, might exhibit higher RNA degradation rates than viable immune cell fractions, might furthermore be unreliably approximated by their respective cellular proportions. However, linearity-based deconvolution on transcriptome data has proven before to provide valuable information [36, 40], so we expect the assumption to approximately hold true.

Wherever linearity is assumed, collinearity between predictors can cause problems. Given a large enough number of biological replicates, this is unlikely to occur for sputum differential cell counts. However, the biological interconnectedness of transcription and cell proportions (imagine a cytokine being strongly upregulated in one cell type and thereby enhancing attraction, proliferation or diapedesis of a second cell type with consequently increased presence) exhibits a further potential for transcript levels correlating with “wrong” cell types. Since the measured outcomes of transcriptional regulation (expression level) and chemotaxis (cell count) vary by different rates and relative, not absolute, cell counts are used as predictor in the linear regression, the risk of this resulting in false positives or negatives in deconvolved data can be seen as minor. Here, biological replication again benefits the accuracy of the deconvolution process.

Simultaneously, fulfilling the requirement of linearity for deconvolution comes with statistical compromises that investigators should be aware of: Performing methylation analysis on beta values instead of the logit-transformed counterpart, M values, causes heteroscedasticity [41] which has to be considered when applying parametric statistical testing procedures. This extends to expression data that are not $\log_2$-transformed. In addition, the assumption of normality is likely violated in both cases. A consequent workaround for these statistical limitations can be permutative testing, as it had been implemented before in the *csSAM* package [40] for expression deconvolution (the package has not been maintained lately and is outdated by now). However, as permutative testing comes with additional limitations itself, we decided to stay with parametric testing due to computational simplicity, speed and reproducibility whilst setting strict significance cutoffs. In any case, significance cutoffs should be chosen carefully and evaluated regarding their respective performance in the analyzed data. The relatively low number of genes that we identified to be simultaneously regulated on both the methylation and transcription level could therefore be caused by applying overly strict cutoffs. Nevertheless, future evaluation of deconvolution performance and determination of a best practice has to take place in larger data sets. Some limitations in this context, such as the limited dynamic range of microarrays or fold change compression, are meanwhile likely to resolve with application of sequencing-based methods.

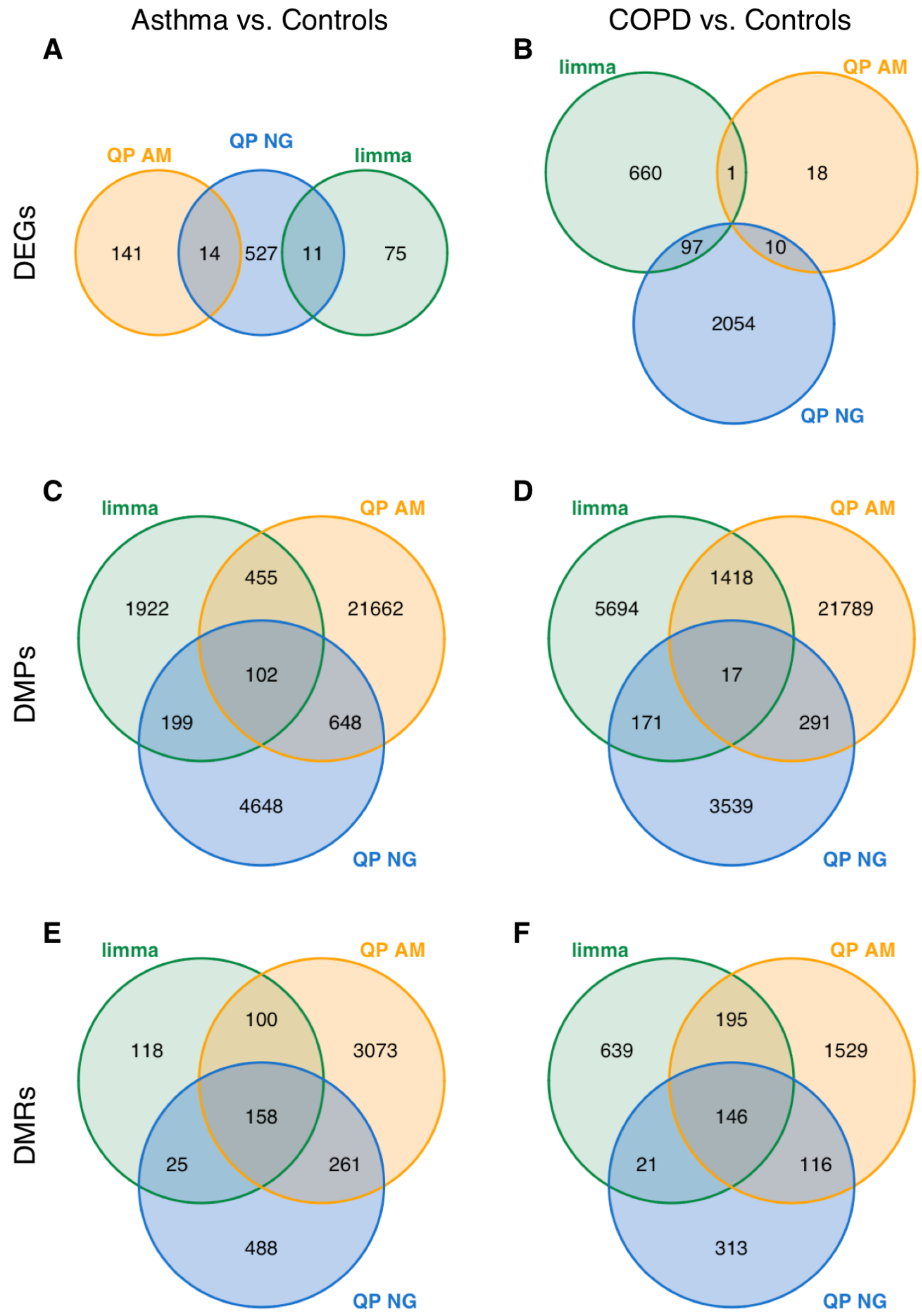

Figure S14: Venn diagram visualizations of differential expression and methylation analysis results before and after deconvolution: DEGs (A, B), DMPs (C, D) and genes associated with DMRs (E, F). Results of both comparisons, asthma vs. controls (A, C, E) and COPD vs. controls (B, D, F), are shown.
limma: conventional group comparison on mixed cell data (via the *limma* package). QP AM/NG: differential expression/methylation analysis on estimates after deconvolution by quadratic programming (QP), for alveolar macrophages (AM) and neutrophils (NG), respectively.

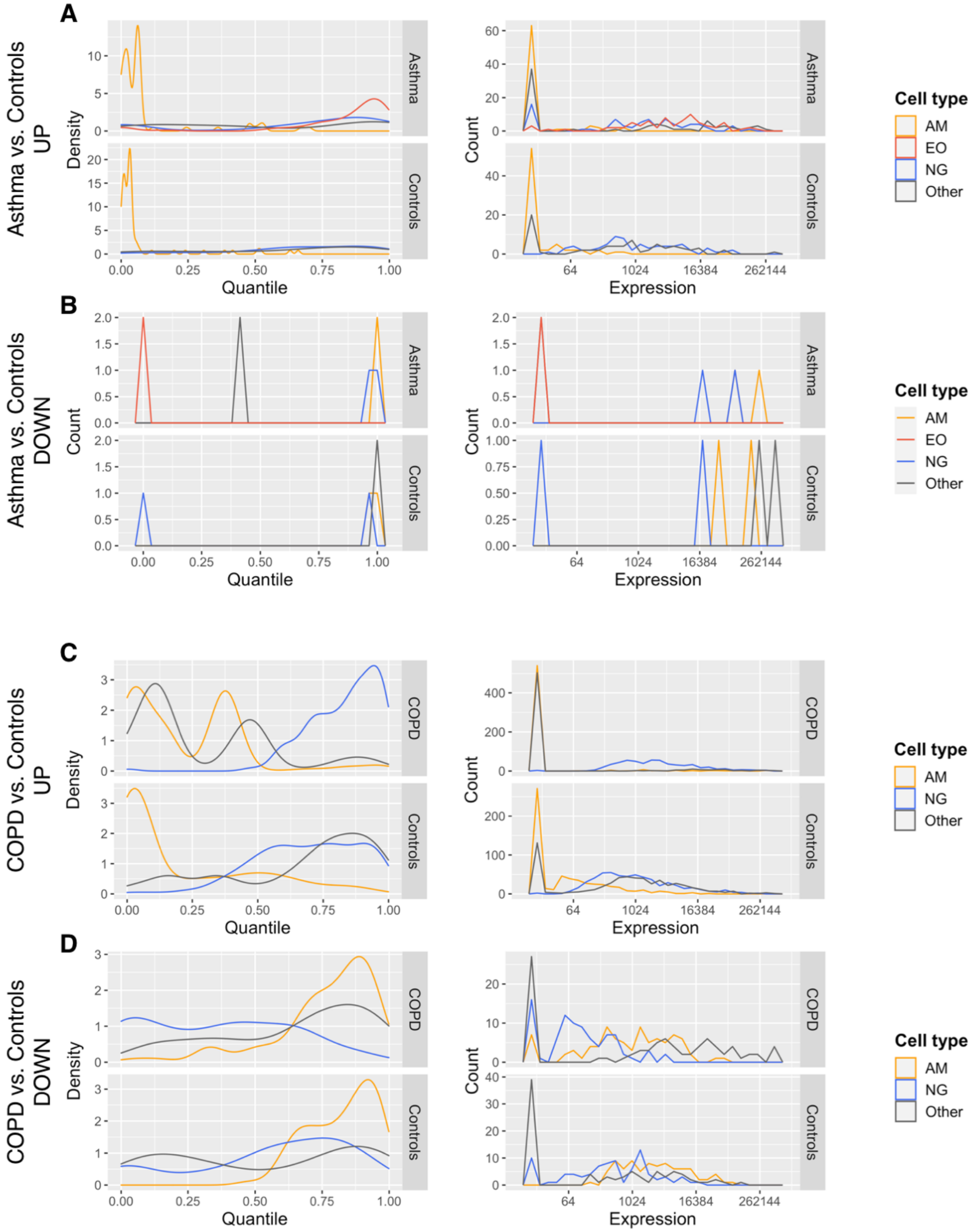

Figure S15: Deconvolved expression values and quantile ranks among estimated expression values of those transcripts that had been identified as DEGs in the mixed-cell analysis only (and not after QP deconvolution), upregulated (A) or downregulated (B) in asthma or upregulated (C) or downregulated (D) in COPD.
AM: alveolar macrophages. NG: neutrophil granulocytes. EO: eosinophils. Other: Sum of residual cell types (weighed intercept).
Of those transcripts identified to be differentially expressed in the asthma group, 12.8 % were still found after deconvolution by quadratic programming (QP). For the COPD group, the rate was 12.9 %. However, only 1.6 and 4.5 % of DEGs found after QP deconvolution were identified in the mixed-cell analyses, respectively (see also Figure S13).

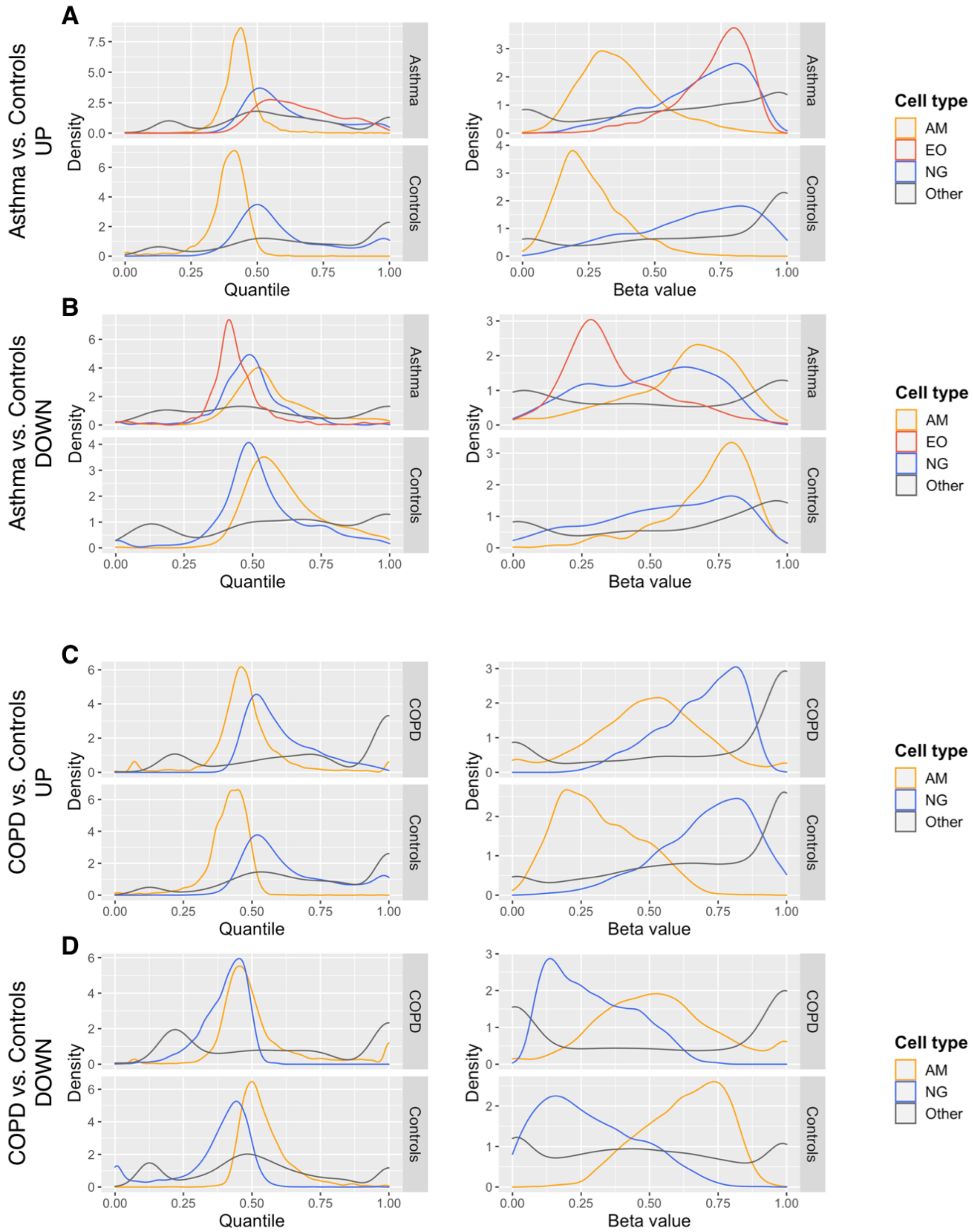

Figure S16: Deconvolved methylation beta values and quantile ranks among estimated beta values of those CpGs that had been identified as DMPs in the mixed-cell analysis only (and not after QP deconvolution), hypermethylated (A) or hypomethylated (B) in asthma or hypermethylated (C) or hypomethylated (D) in COPD. AM: alveolar macrophages. NG: neutrophil granulocytes. EO: eosinophils. Other: Sum of residual cell types (weighed intercept).
As the overall distribution of beta values follows a bipolar pattern (see also Figure S6), the reader might find beta values easier to interpret than quantile ranks in this context.
Of those DMPs found by mixed-cell analysis in the asthma group, 28.2 % were still identified after deconvolution by quadratic programming (QP). For the COPD group, the rate was 22.2 %. However, only 2.7 and 5.9 % of DMPs found after QP deconvolution were identified in the mixed-cell analyses, respectively (see also Figure S13).

Enrichment results

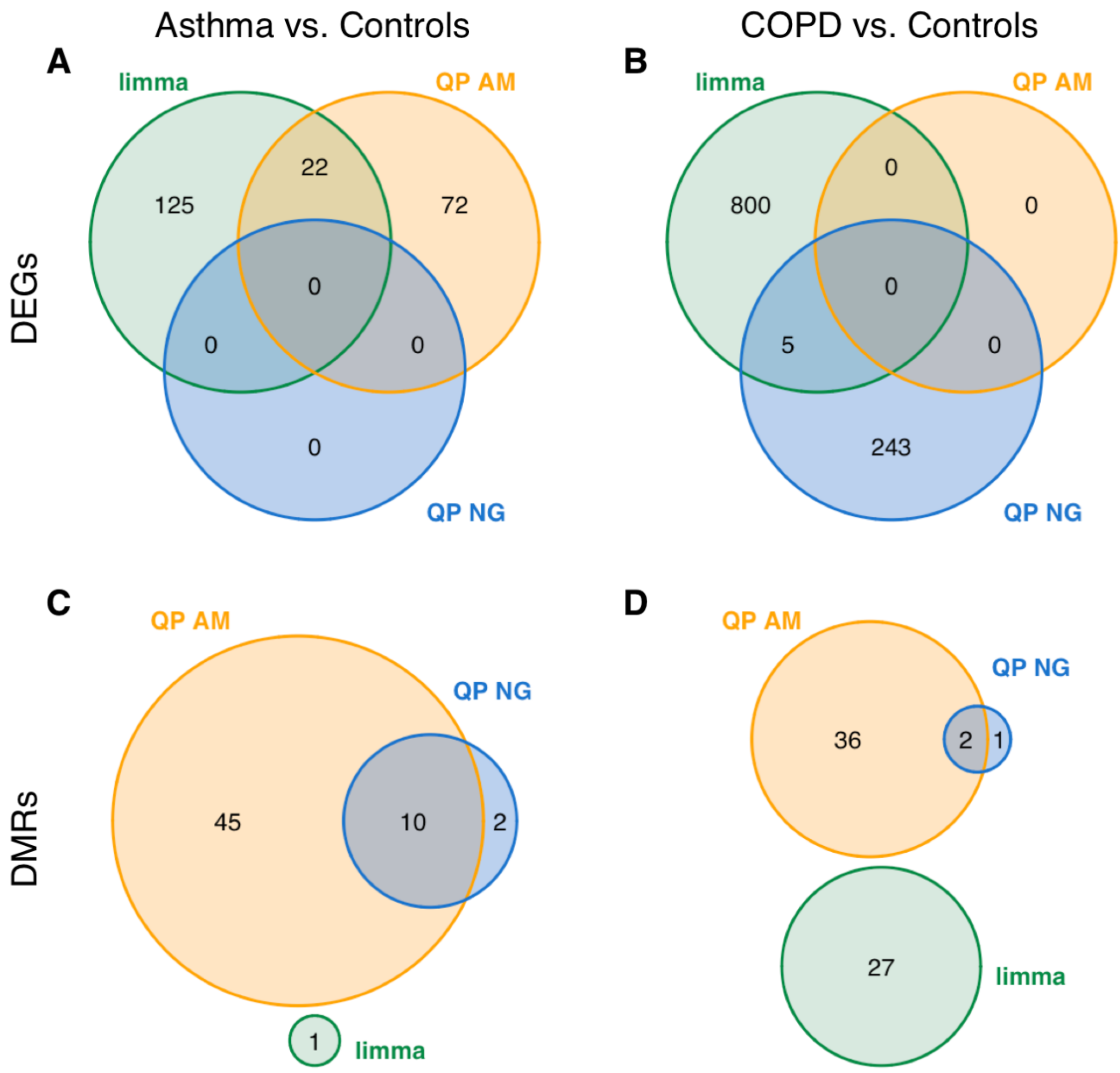

Figure S17: Venn diagram visualizations of Gene Ontology terms enriched in DEGs (A, B) and DMRs (C, D) in asthma (A, C) and COPD (B, D).

limma: conventional group comparison on mixed cell data (via the *limma* package). QP AM/NG: differential expression/methylation analysis on estimates after deconvolution by quadratic programming (QP), for alveolar macrophages (AM) and neutrophils (NG), respectively.

Integrative analysis

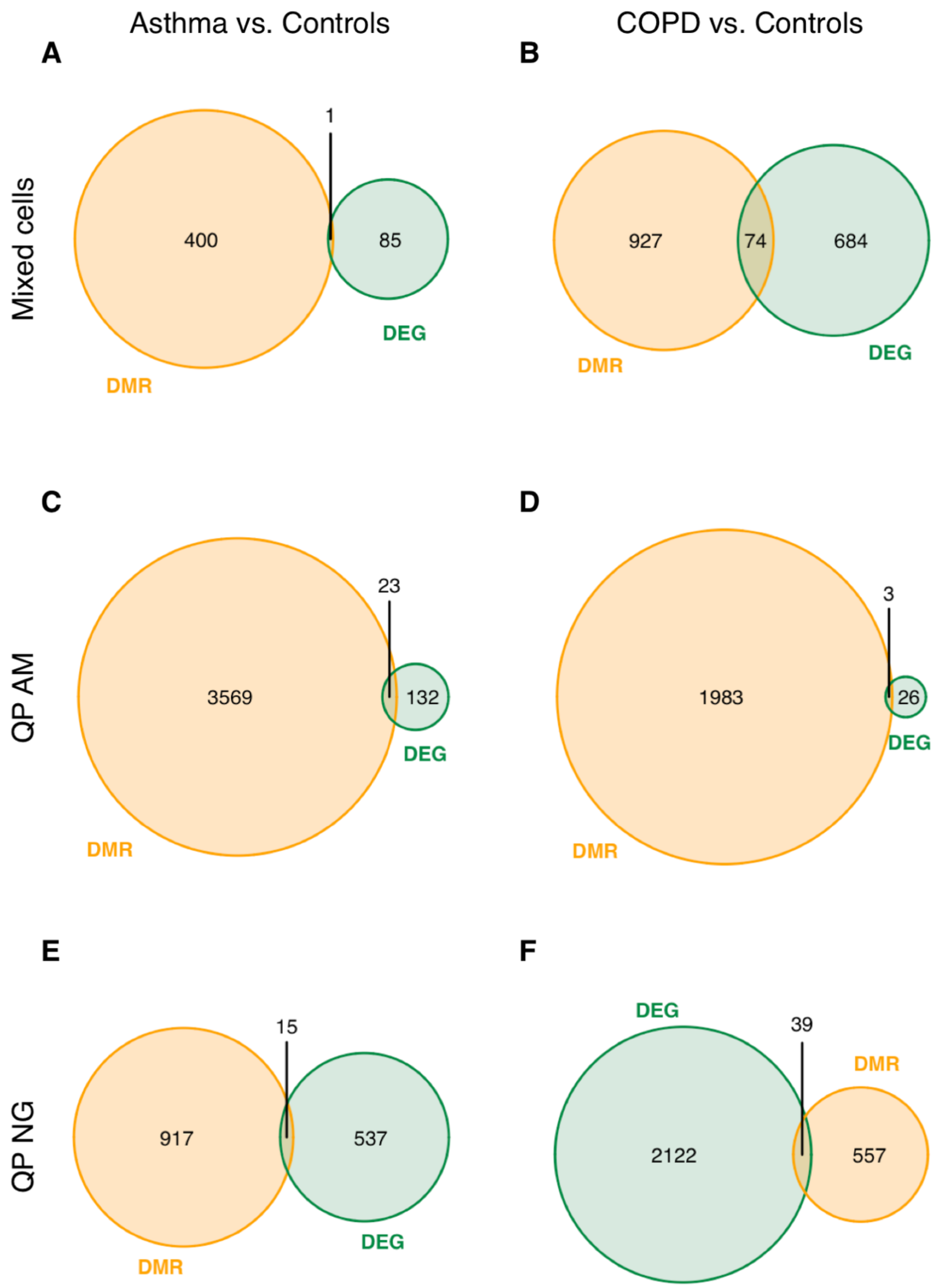

Figure S18: Venn diagram visualizations of DEGs and DMR-associated genes in asthma (A, C, E) and COPD (B, D, F) as identified via the analysis of mixed-cell data (A, B) and deconvolved cell type-specific data for macrophages (C, D) and neutrophils (E, F).
QP AM/NG: differential expression/methylation analysis on estimates after deconvolution by quadratic programming (QP), for alveolar macrophages (AM) and neutrophils (NG), respectively.

Data comparison

In a search for publicly available omics data sets generated from sputum or BAL on which we could validate our deconvolution's performance, we accessed the Gene Expression Omnibus (GEO) database (https://www.ncbi.nlm.nih.gov/geo/, accessed on 21 August 2020). We used the search strings "sputum asthma" (19 series hits), "BAL asthma" (25 series hits), "sputum COPD" (6 series hits) and "BAL COPD" (8 series hits) and checked the series hits manually for suitability. After excluding studies performed in mice, studies that did not provide data from sputum or BAL cells and studies that were conducted on purified cell samples, none of the remaining data sets was suitable to be supplied to our deconvolution approach due to missing information about the cellular composition of individual samples (although differential cell counts had been performed as derived from the publications associated with the data sets, e.g. [42]). Only one study provided detailed sputum differential cell counts with the omics data [43], however, the transcriptomic analysis was performed on purified macrophages and not on mixed-cell samples in this case.

We further screened the encountered data and associated publications for differential transcription analyses performed on purified cell types to which we could compare our results. In [44], alveolar macrophages were purified from asthmatics and checked for differential expression against healthy controls. However, the sample size of the study was very low (n = 5 per group), some asthmatics received antihistamines whilst none of the subjects needed therapy with inhaled corticosteroids (as opposed to our study, indicating that the overall asthma phenotype was milder) and, according to the manuscript, the authors did not apply a statistical correction for multiple testing to their results. Strictly speaking, we did not observe an appreciable overlap between the reported DEGs and DEGs identified in our analysis, but considering the aforementioned points, we don't interpret this as a falsification of our results. In [43] and [45], analyses were performed on purified macrophages from COPD patients. Since we were unfortunately not able to reliably estimate COPD macrophage profiles in our small data set, we refrained from comparing the reported findings to our results. In [46], a differential expression analysis comprising purified sputum macrophages in both asthma and COPD is presented. However, the purity of macrophages in the analyzed samples (a cutoff of > 50 % was applied by the authors) is rather low so we consider the data not macrophage-specific.

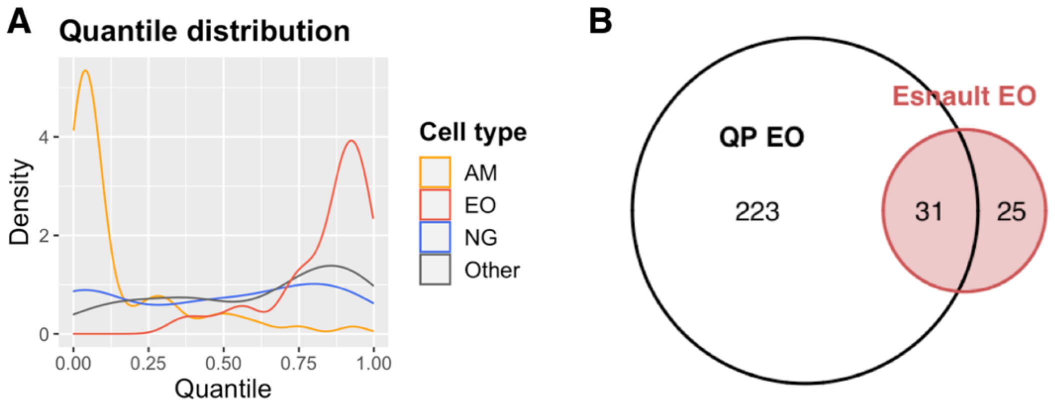

Figure S19: Quantile ranks of genes of the eosinophil set defined by Esnault et al. amongst the deconvolved expression estimates of each cell type (A) and overlap between the Esnault eosinophil gene set and genes differentiating eosinophils from macrophages and neutrophils in our data after deconvolution (B).
AM: alveolar macrophages. NG: neutrophil granulocytes. EO: eosinophils. Other: Sum of residual cell types (weighed intercept). QP: deconvolution by quadratic programming.

Esnault et al. [47] defined a core set of 57 genes predominantly expressed by asthma eosinophils through transcriptome analyses in BAL and sputum in the context of allergen challenges which they validated in purified lung eosinophils subsequently. From these 57 genes, 56 were present in our expression data set after the initial data processing steps. The distribution of their respective quantile ranks among the expression estimates is shown in Figure S19A for each deconvolved cell type. Corresponding to the findings by Esnault et al., we observed the gene set to be primarily expressed in eosinophils.

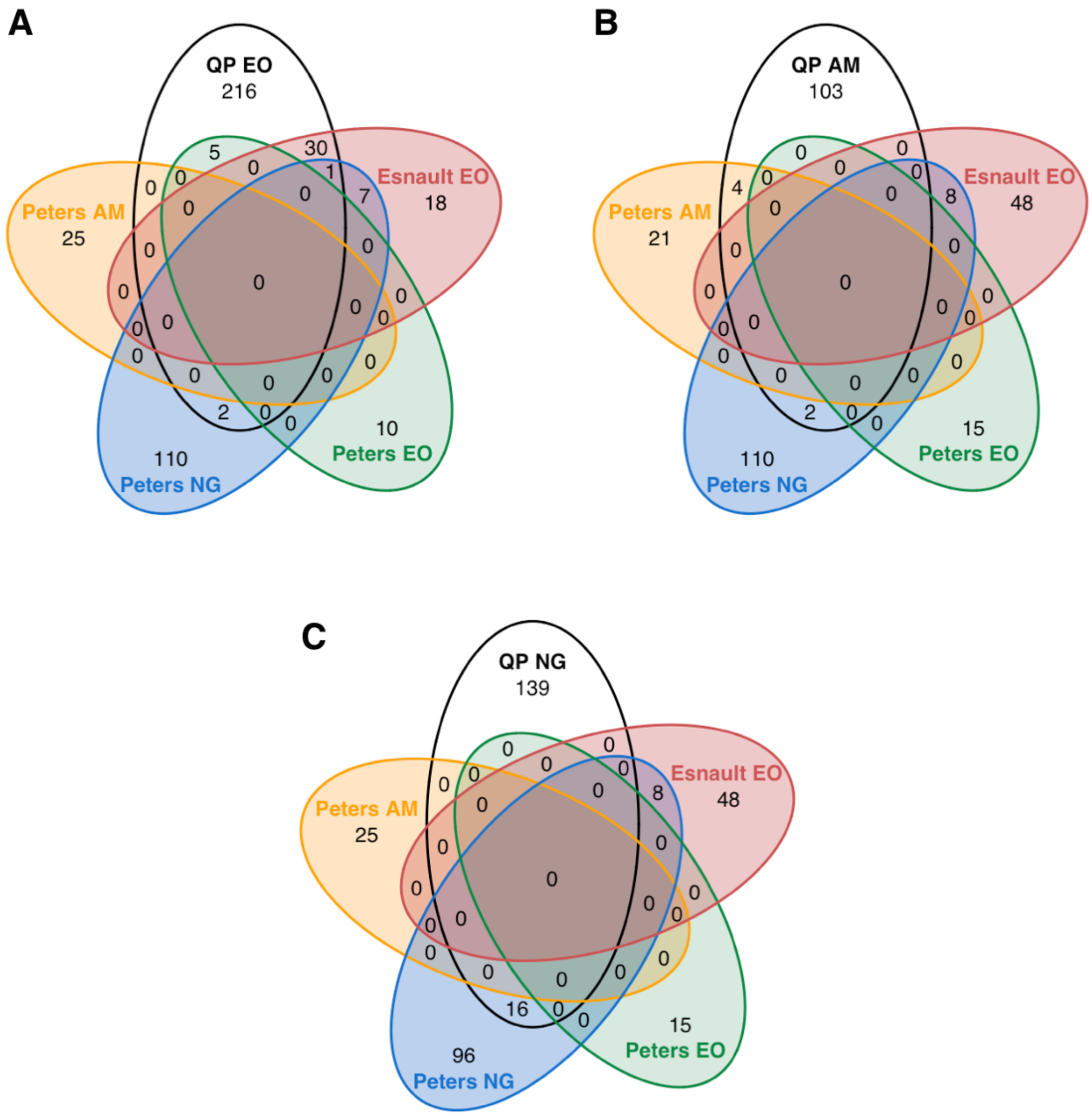

Figure S20: Overlaps between gene sets discriminating eosinophils (A), macrophages (B) and neutrophils (C) in the deconvolved asthma expression data and the eosinophil gene set defined by Esnault et al. as well as cell type-specific gene sets defined by Peters et al.
AM: alveolar macrophages. NG: neutrophil granulocytes. EO: eosinophils. QP: deconvolution by quadratic programming.

We further specified gene sets that differentiated eosinophils, neutrophils and macrophages in our deconvolved asthma expression data. To be associated with one of the cell types, genes had to exhibit a (positive) $\log_2 FC > 2$ in relation to both of the other estimated cell types at a BH-corrected (one-sided)

p value < 0.05. Note that in this constellation, statistical testing does not necessarily give meaningful results since the deconvolved estimates are derived from the same original data. Concordantly, the fold change cutoff was observed to be the predominant criterion for selection. Please note further that under these circumstances, the individual genes are not truly cell-specific (cell-exclusive) but rather estimated to be expressed at a higher level in one of the considered cell types than in both of the others. By this means, 31 out of 56 genes of the Esnault et al. gene set were found to discriminate eosinophils in our deconvolved estimates (see Figure S19B). In light of the multifaceted experimental setup employed by Esnault et al., including allergen challenges as well as application of the anti-IL5 antibody mepolizumab, we consider this to be a rather good overlap with our data of "steady state" eosinophils (no therapeutic/experimental intervention in our study).

Peters et al. previously derived cell-specific gene sets from transcriptome data that had been generated from blood and bone marrow samples and used these cell type-specific gene sets in the analysis of the sputum transcriptome in asthma [48]. Including these gene sets into our comparison, we observed predominant overlaps of our discriminating gene sets with the according cell types in the data by Peters et al. (see Figure S20). Furthermore, we observed that the eosinophil expression data provided by Esnault et al. might have been contaminated by gene expression from neutrophils in the context of the described allergen challenges (see overlap in Figure S20C). Otherwise, this implies that gene expression profiles of blood cells differ from those of cells of the same type in the lung environment. Further discussion concerning this issue is presented in the main manuscript.

References

1. Pizzichini, M.M.M., et al., *Safety of sputum induction.* European Respiratory Journal, 2002. **20**(37 suppl): p. 9s.

2. Weiszhar, Z. and I. Horvath, *Induced sputum analysis: step by step.* Breathe, 2013. **9**(4): p. 300.

3. R Core Team (2019). *R: A language and environment for statistical computing.* Available from: https://www.R-project.org/

4. Wickham, H., J. Hester, and W. Chang (2020). *devtools: Tools to Make Developing R Packages Easier. R package version 2.2.2.* Available from: https://CRAN.R-project.org/package=devtools

5. Huber, W., et al., *Orchestrating high-throughput genomic analysis with Bioconductor.* Nat Methods, 2015. **12**(2): p. 115-21.

6. Ritchie, M.E., et al., *limma powers differential expression analyses for RNA-sequencing and microarray studies.* Nucleic Acids Res, 2015. **43**(7): p. e47.

7. Aryee, M.J., et al., *Minfi: a flexible and comprehensive Bioconductor package for the analysis of Infinium DNA methylation microarrays.* Bioinformatics, 2014. **30**(10): p. 1363-9.

8. Hansen, K.D. (2016). *IlluminaHumanMethylation450kanno.ilmn12.hg19: Annotation for Illumina's 450k methylation arrays. R package version 0.6.0.*

9. Hansen, K.D. and M. Aryee (2012). *IlluminaHumanMethylation450kmanifest: Annotation for Illumina's 450k methylation arrays. R package version 0.4.0.*

10. Neuwirth, E. (2014). *RColorBrewer: ColorBrewer Palettes. R package version 1.1-2.* Available from: https://CRAN.R-project.org/package=RColorBrewer

11. Bengtsson, H. (2020). *matrixStats: Functions that Apply to Rows and Columns of Matrices (and to Vectors). R package version 0.56.0.* Available from: https://CRAN.R-project.org/package=matrixStats

12. Peters, T.J., et al., *De novo identification of differentially methylated regions in the human genome.* Epigenetics Chromatin, 2015. **8**: p. 6.

13. Peters, T. (2020). *DMRcatedata: Data Package for DMRcate. R package version 2.2.1.*

14. S original by Berwin A. Turlach, R port by Andreas Weingessel <Andreas.Weingessel@ci.tuwien.ac.at>, and Fortran contributions from Cleve Moler dpodi/LINPACK (2019). *quadprog: Functions to Solve Quadratic Programming Problems. R package version 1.5-8.* Available from: https://CRAN.R-project.org/package=quadprog

15. Warnes, G.R., B. Bolker, and T. Lumley (2018). *gtools: Various R Programming Tools. R package version 3.8.1.* Available from: https://CRAN.R-project.org/package=gtools

16. Arnholt, A.T. and B. Evans (2017). *BSDA: Basic Statistics and Data Analysis.* Available from: https://github.com/alanarnholt/BSDA, https://alanarnholt.github.io/BSDA/

17. Wickham, H., *The Split-Apply-Combine Strategy for Data Analysis.* Journal of Statistical Software, 2011. **40**(1): p. 29.

18. Yu, G., et al., *clusterProfiler: an R package for comparing biological themes among gene clusters.* Omics, 2012. **16**(5): p. 284-7.

19. Carlson, M. (2019). *org.Hs.eg.db: Genome wide annotation for Human. R package version 3.10.0.* Available from: https://bioconductor.org/packages/release/data/annotation/html/org.Hs.eg.db.html

20. Dolgalev, I. (2019). *msigdbr: MSigDB Gene Sets for Multiple Organisms in a Tidy Data Format. R package version 7.0.1.* Available from: https://CRAN.R-project.org/package=msigdbr

21. Wickham, H. and L. Henry (2020). *tidyr: Tidy Messy Data. R package version 1.0.2.* Available from: https://CRAN.R-project.org/package=tidyr

22. Wickham, H., *ggplot2: Elegant Graphics for Data Analysis*. 2016: Springer-Verlag New York. Available from: https://ggplot2.tidyverse.org

23. Wilke, C.O. (2019). *cowplot: Streamlined Plot Theme and Plot Annotations for 'ggplot2'. R package version 1.0.0.* Available from: https://CRAN.R-project.org/package=cowplot

24. Chen, H. (2018). *VennDiagram: Generate High-Resolution Venn and Euler Plots. R package version 1.6.20.* Available from: https://CRAN.R-project.org/package=VennDiagram

25. Onuchic, V. (2019). *EDec: Cell type specific analysis of complex tissues through Epigenomic Deconvolution. R package version 0.9.* Available from: https://github.com/BRL-BCM/EDec

26. Abdel-Aziz, M.I., et al., *Omics for the future in asthma.* Semin Immunopathol, 2020. **42**(1): p. 111-126.

27. Auffray, C., et al., *An integrative systems biology approach to understanding pulmonary diseases.* Chest, 2010. **137**(6): p. 1410-6.

28. Peters, M.C., et al., *Measures of gene expression in sputum cells can identify T2-high and T2-low subtypes of asthma.* J Allergy Clin Immunol, 2013.

29. Hrdlickova, R., M. Toloue, and B. Tian, *RNA-Seq methods for transcriptome analysis.* Wiley Interdiscip Rev RNA, 2017. **8**(1).

30. Opitz, L., et al., *Impact of RNA degradation on gene expression profiling.* BMC Med Genomics, 2010. **3**: p. 36.

31. Gallego Romero, I., et al., *RNA-seq: impact of RNA degradation on transcript quantification.* BMC Biol, 2014. **12**: p. 42.

32. Liu, X., et al., *Normalization Methods for the Analysis of Unbalanced Transcriptome Data: A Review.* Front Bioeng Biotechnol, 2019. **7**: p. 358.

33. Bolstad, B.M., et al., *A comparison of normalization methods for high density oligonucleotide array data based on variance and bias.* Bioinformatics, 2003. **19**(2): p. 185-93.

34. Cohen, J., *A power primer.* Psychol Bull, 1992. **112**(1): p. 155-9.

35. Onuchic, V., et al., *Epigenomic Deconvolution of Breast Tumors Reveals Metabolic Coupling between Constituent Cell Types.* Cell Rep, 2016. **17**(8): p. 2075-2086.

36. Gong, T., et al., *Optimal deconvolution of transcriptional profiling data using quadratic programming with application to complex clinical blood samples.* PLoS One, 2011. **6**(11): p. e27156.

37. Goldfarb, D. and A. Idnani, *A numerically stable dual method for solving strictly convex quadratic programs.* Mathematical Programming, 1983. **27**(1): p. 1-33.

38. Cohen, A., *Comparing Regression Coefficients Across Subsamples: A Study of the Statistical Test.* Sociological Methods & Research, 1983. **12**(1): p. 77-94.

39. Dallas, P.B., et al., *Gene expression levels assessed by oligonucleotide microarray analysis and quantitative real-time RT-PCR -- how well do they correlate?* BMC Genomics, 2005. **6**: p. 59.

40. Shen-Orr, S.S., et al., *Cell type-specific gene expression differences in complex tissues.* Nat Methods, 2010. **7**(4): p. 287-9.

41. Du, P., et al., *Comparison of Beta-value and M-value methods for quantifying methylation levels by microarray analysis.* BMC Bioinformatics, 2010. **11**: p. 587.

42. Kuo, C.S., et al., *T-helper cell type 2 (Th2) and non-Th2 molecular phenotypes of asthma using sputum transcriptomics in U-BIOPRED.* Eur Respir J, 2017. **49**(2).

43. Morrow, J.D., et al., *RNA-sequencing across three matched tissues reveals shared and tissue-specific gene expression and pathway signatures of COPD.* Respir Res, 2019. **20**(1): p. 65.

44. Madore, A.M., et al., *Alveolar macrophages in allergic asthma: an expression signature characterized by heat shock protein pathways.* Hum Immunol, 2010. **71**(2): p. 144-50.

45. O'Beirne, S.L., et al., *Alveolar Macrophage Immunometabolism and Lung Function Impairment in Smoking and Chronic Obstructive Pulmonary Disease.* Am J Respir Crit Care Med, 2020. **201**(6): p. 735-739.

46. Paplińska-Goryca, M., et al., *Genetic characterization of macrophages from induced sputum of patients with asthma and chronic obstructive pulmonary disease.* Pol Arch Intern Med, 2018. **128**(9): p. 559-562.

47. Esnault, S., et al., *Identification of genes expressed by human airway eosinophils after an in vivo allergen challenge.* PLoS One, 2013. **8**(7): p. e67560.

48. Peters, M.C., et al., *A Transcriptomic Method to Determine Airway Immune Dysfunction in T2-High and T2-Low Asthma.* Am J Respir Crit Care Med, 2019. **199**(4): p. 465-477.
